# Vector competence of *Aedes albopictus* field populations from Reunion Island exposed to local epidemic dengue viruses

**DOI:** 10.1371/journal.pone.0310635

**Published:** 2024-09-19

**Authors:** Sarah Hafsia, Tatiana Barbar, Haoues Alout, Fiona Baudino, Cyrille Lebon, Yann Gomard, David A. Wilkinson, Toscane Fourié, Patrick Mavingui, Célestine Atyame

**Affiliations:** 1 Université de La Réunion, UMR PIMIT (Processus Infectieux en Milieu Insulaire Tropical), CNRS 9192, INSERM 1187, IRD 249, La Réunion, France; 2 Unité Mixte de Recherche Animal Santé Territoires Risques Écosystèmes, F-34398, CIRAD/INRAE/Université de Montpellier, Université de Montpellier, Montpellier, France; Instituto Nacional de Salud Pública, MEXICO

## Abstract

Dengue virus (DENV) is the most prevalent mosquito-borne *Flavivirus* that affects humans worldwide. *Aedes albopictus*, which is naturally infected with the bacteria *Wolbachia*, is considered to be a secondary vector of DENV. However, it was responsible for a recent DENV outbreak of unprecedented magnitude in Reunion Island, a French island in the South West Indian Ocean. Moreover, the distribution of the cases during this epidemic showed a spatially heterogeneous pattern across the island, leading to questions about the differential vector competence of mosquito populations from different geographic areas. The aim of this study was to gain a better understanding of the vector competence of the *Ae*. *albopictus* populations from Reunion Island for local DENV epidemic strains, while considering their infection by *Wolbachia*. Experimental infections were conducted using ten populations of *Ae*. *albopictus* sampled across Reunion Island and exposed to three DENV strains: one strain of DENV serotype 1 (DENV-1) and two strains of DENV serotype 2 (DENV-2). We analyzed three vector competence parameters including infection rate, dissemination efficiency and transmission efficiency, at different days post-exposition (dpe). We also assessed whether there was a correlation between the density of *Wolbachia* and viral load/vector competence parameters. Our results show that the *Ae*. *albopictus* populations tested were not able to transmit the two DENV-2 strains, while transmission efficiencies up to 40.79% were observed for the DENV-1 strain, probably due to difference in viral titres. Statistical analyses showed that the parameters mosquito population, generation, dpe and area of sampling significantly affect the transmission efficiencies of DENV-1. Although the density of *Wolbachia* varied according to mosquito population, no significant correlation was found between *Wolbachia* density and either viral load or vector competence parameters for DENV-1. Our results highlight the importance of using natural mosquito populations for a better understanding of transmission patterns of dengue.

## Introduction

Dengue is the most widespread mosquito-borne disease affecting humans, with half of the global population located in 128 tropical and subtropical countries, at risk of infection [1,2]. Most of the 96 million symptomatic cases per year are dengue fevers, which are characterized by a panel of mild symptoms [3]. However, some cases demonstrate much more severe syndromes called severe dengue (which can include severe plasma leakage, severe hemorrhagic syndrome, with or without shock, and various organ dysfunctions). Severe dengue is responsible for 20,000 deaths each year [4,5]. The etiological agent of the disease, the dengue virus (DENV), is a positive single-stranded linear RNA genome (11 kilobases), belonging to the genus *Flavivirus* (*Flaviviridae* family). Four different DENV serotypes exist, each of which is divided into several genotypes based on molecular analyses [6–8]. Long term serotype-specific immunity follows a DENV infection and lasts for several decades [9], whereas secondary infection with a heterologous serotype can lead to severe disease manifestations through the antibody-dependent enhancement effect [10,11]. The DENV is transmitted to humans through the bite of infected female mosquitoes, mostly of the *Aedes* genus. Globally, the mosquito species *Aedes aegypti* is considered as the primary DENV vector, with *Aedes albopictus* recognized as a secondary vector [5,12–14]. However, outbreaks of dengue involving *Ae*. *albopictus* have been reported in some countries, such as in Japan (in 1942 and in 2014) [15,16], Hawaii (in 2001) [17], Madagascar (in 2006) [18], Gabon (in 2007) [19], China (in 2004 and 2010) [20,21] and more recently in Reunion Island (in 2017) [22].

Reunion Island is an overseas French department located in South-Western Indian Ocean (SWIO), about 700 km east of Madagascar. Several dengue outbreaks have been documented on the island, with the first officially recorded one dating back to 1977 [23]. Subsequently, low-intensity outbreaks have been reported for several years, with less than 230 cases per year and no death related to dengue. However, Reunion Island has faced unprecedented epidemiological patterns from 2017 to 2021. During this period, 71,636 confirmed cases, 542 severe forms and 78 deaths were reported [22]. All four DENV serotypes have been detected in Reunion Island since 1977: the three serotypes DENV-1, DENV-2 and DENV-3 in autochthonous cases, and all four serotypes in imported cases [22]. A strong seasonal pattern in dengue incidence has been reported on the island, with peaks occurring between March and June (i.e., during the hot and rainy season) [22]. Besides this seasonality, the geographic distribution of dengue cases is often heterogeneous across the island, with the western and the southern parts being the most affected compared to the northern, eastern and central parts [22]. Although factors related to human populations (demography, geographic mobility, immunity) and DENV genetics may explain the transmission pattern of DENV in Reunion Island, the role of mosquito populations should also be taken into account [22].

Among the 12 species of mosquitoes encountered in Reunion Island, both *Ae*. *aegypti* and *Ae*. *albopictus* are present [24–26]. However, *Ae*. *albopictus* is the most abundant mosquito species and is commonly found throughout the island, even at altitudes exceeding 1,000 m [24,27]. This species was identified as the main vector responsible for a significant Chikungunya outbreak on Reunion Island in 2005–2006 [28], and it is also a major vector involved in dengue epidemics on the island [23,29–31]. The role of *Ae*. *albopictus* in the transmission of DENV in Reunion Island is supported by its large distribution across the island and the occurrence of peaks of DENV transmission that coincide with periods of high population densities of this vector [32,33]. DENV-1 was detected in pooled samples of *Ae*. *albopictus* collected in Reunion Island during the 2004 epidemic [30]. Other studies have shown that *Ae*. *albopictus* from Reunion Island are capable of being infected with both DENV-1 [34] and DENV-2 [35,36]. However, these data do not provide a clear picture of the vector competence variability (the ability of a vector to be infected and to transmit a pathogen) among *Ae*. *albopictus* populations from Reunion Island and their potential role in the geographical contrasts of dengue cases.

The aim of this study was to examine vector competence of natural populations of *Ae*. *albopictus* from Reunion Island, collected in areas of high and low DENV transmission. A laboratory line of *Ae*. *aegypti* from Reunion Island was used as a control. Experimental infections were performed using local epidemic DENV-1 and DENV-2 strains. Since no geographic structure of the genetic diversity of *Ae*. *albopictus* populations has been observed in Reunion Island [37,38], we also assessed the influence of the density of endosymbiotic bacteria *Wolbachia* on vector competence phenotypes. *Aedes albopictus* is naturally infected with two *Wolbachia* strains, namely *w*AlbA and *w*AlbB [39], which can interfere with the replication and transmission of DENV [35,40,41]. The results of this investigation may help to better understand the role of *Ae*. *albopictus* populations in the epidemiological patterns of dengue in Reunion Island.

## Materials and methods

### Mosquitoes

**Fig 1:**
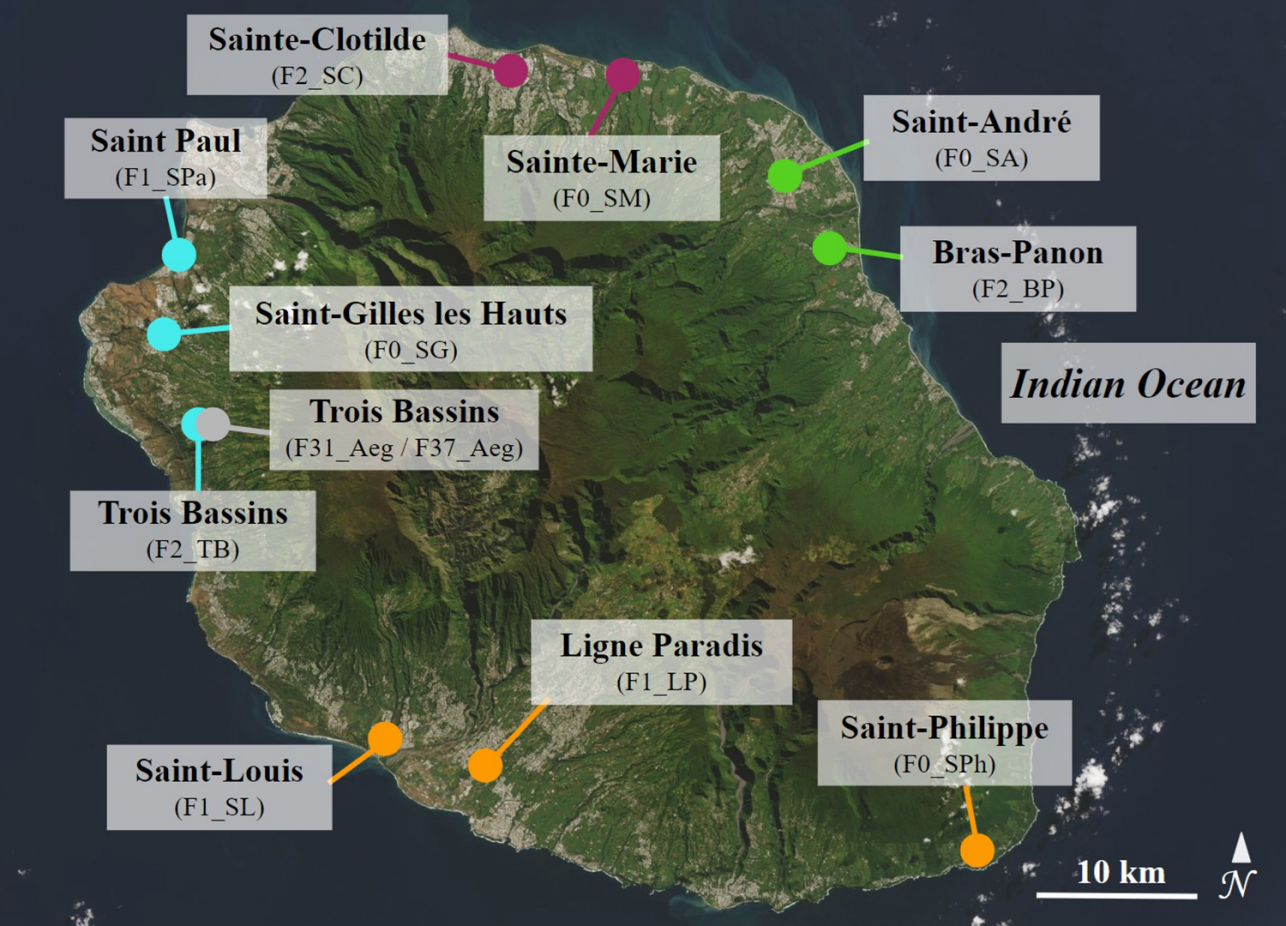
Map of sampling sites of *Aedes albopictus* and *Aedes aegypti* in Reunion Island. For *Ae*. *albopictus*, the sampling sites are colored according to geographic regions: Orange, South; blue, West; purple, North; and green, East. The unique *Ae*. *aegypti* population is colored in gray. Population codes and the generation (i.e. F_0_, F_1_, F_2_, F_31_, or F_37_ generation) at which mosquitoes were used for the vector competence experiments are given in brackets. The satellite image was extracted from the NASA Earth Observatory public domain image database [42].

*Aedes albopictus* specimens were collected as eggs, larvae and pupae in 10 localities across Reunion Island in 2020 and 2021: Sainte-Marie (F0_SM), Saint-André (F0_SA), Saint-Gilles les Hauts (F0_SG), Saint-Philippe (F0_SPh), Saint-Paul (F1_SPa), Saint-Louis (F1_SL), Ligne Paradis (F1_LP), Sainte-Clotilde (F2_SC), Bras-Panon (F2_BP) and Trois Bassins (F2_TB) (Fig 1 and Table 1). Field samples (F_0_ generation) were reared in the laboratory under standard conditions [26 ± 1°C, 80% relative humidity (RH), 12 h light/12 h dark photoperiod]. Larvae were fed with yeast tablets, and adults were provided with 10% sucrose solution. Samples collected as eggs were amplified for one generation (F_1_) or two generations (F_2_) before vector competence experiments. To achieve this, adult females were artificially fed with bovine blood using the Hemotek feeding system (Hemotek Limited, Great Harwood, UK) covered by pig intestine. Experimental infections were conducted using four *Ae*. *albopictus* populations of F_0_ generation (F0_SM, F0_SA, F0_SG and F0_SPh), three populations of F_1_ generation (F1_SPa, F1_SL and F1_LP), and three populations of F_2_ generation (F2_SC, F2_BP and F2_TB) (Table 1). A laboratory colony of *Ae*. *aegypti* (F_31_ and F_37_ generations), established from mosquitoes collected in the Trois Bassins locality in 2014 (F31_Aeg and F37_Aeg), was also used (Fig 1 and Table 1).

**Table 1:** General information about mosquito populations and DENV strains used in this study. **For each population and condition, the number of samples examined after DENV exposure are indicated.** dpe = days post-exposition.

Mosquitoes					DENV strain	Number of samples examined		
Species	Sampling site (population code)	Geographic area	Generation	Date of collection		14 dpe	21 dpe	28 dpe
***Aedes albopictus***	Sainte-Marie **(F0_SM)**	North	F_0_	April 2021	DENV-1	32	48	48
				May 2021	DENV-2_EVAg	48	48	48
					DENV-1	24	24	26
	Sainte-Clotilde **(F2_SC)**	North	F_2_	February to March 2020	DENV-1	32	38	0
	Saint-Gilles les Hauts **(F0_SG)**	West	F_0_	April 2021	DENV-1	32	48	48
					DENV-2_ EVAg	0	0	5
	Saint-Paul **(F1_SPa)**	West	F_1_	February to March 2020	DENV-2_JUL	17	0	0
	Trois Bassins **(F2_TB)**	West	F_2_	February to March 2020	DENV-1	48	48	76
	Saint-Philippe **(F0_SPh)**	South	F_0_	April 2021	DENV-1	32	48	48
					DENV-2_EVAg	0	0	16
	Saint-Louis **(F1_SL)**	South	F_1_	February to March 2020	DENV-2_JUL	22	0	0
	Ligne Paradis **(F1_LP)**	South	F_1_	February to March 2020	DENV-2_JUL	17	0	0
	Saint-André **(F0_SA)**	East	F_0_	April 2021	DENV-1	32	48	32
					DENV-2_EVAg	48	48	48
	Bras-Panon **(F2_BP)**	East	F_2_	February to March 2020	DENV-1	48	48	70
***Aedes aegypti***	Trois Bassins **(F31_Aeg or F37_Aeg)**	Laboratory colony	F_31_	2014	DENV-2_EVAg	35	0	0
					DENV-2_JUL	32	0	0
			F_37_		DENV-1	32	48	30

### Viral strains

Three clinical DENV strains isolated from autochthonous human cases from Reunion Island were used: one DENV-1 strain of genotype 1, (GenBank accession number: ON631277), and two DENV-2 strains of the same lineage inside the cosmopolitan genotype, DENV-2_JUL (GenBank accession number: MN272404) and DENV-2_EVAg (EVAg reference: UVE/DENV-2/2018/RE/47099). The DENV-1 strain was isolated on Vero E6 cells (ATCC, ref. CRL-1586) from a serum sampled in 2019 [43]. The DENV-2_JUL was isolated on Vero E6 cells from a blood sample collected from a patient in 2018 [44]. The DENV-2_EVAg was purchased as lyophilizate from the European Virus Archive goes global (EVAg) at passage 4. This latest DENV strain was isolated from a traveler returning from Reunion Island to mainland France in 2018. Before viral production, the lyophilized DENV-2_EVAg was resuspended into 200 μl distilled water. Viral stocks of the three DENV strains used in experimental infections were amplified on Vero E6 cells at a MOI of 0.1 in an Eagle’s minimum essential medium (MEM) supplemented with 2% heat-inactivated fetal bovine serum (FBS), 2 mmol/l L-glutamine, 1 mmol/l sodium pyruvate, 10 U/ml of penicillin, 0.1 mg/ml of streptomycin and 0.5 μg/ml of fungizone (PAN Biotech, Aidenbach, Germany). Vero cells were maintained at 37°C with a 5% CO_2_ atmosphere. For all virus stocks, supernatants were harvested three to five days post-infection after the onset of cytopathic effects and then frozen at -80°C until use.

### Experimental infections

Seven to fifteen-day-old female mosquitoes were isolated in small cages (16×16×16 cm) and starved for 24 to 30 hours. After this starvation period, they were allowed to feed for 45 minutes on infectious blood meals consisting of 1 ml of washed rabbit erythrocytes, 1 ml of DENV suspension and 5 mM (21 μl) of adenosine triphosphate used as a phagostimulant. The infectious blood meal was delivered to mosquitoes using the Hemotek feeding system (Hemotek Limited, Great Harwood, UK) covered with pig intestine. Because we were unable to increase the titres of viral stocks of the two DENV-2 strains for experimental infections, infectious blood meals were performed with the maximum possible virus titre which differed between DENV strains with 7×10^6^ PFU/ml, 6.8×10^4^ PFU/ml and 3.2×10^5^ PFU/ml for DENV-1, DENV-2_JUL and DENV-2_EVAg, respectively. Then, mosquitoes were cold-anesthetized, and engorged females were transferred into a climatic chamber (26±1°C, 80% of RH and with a photoperiod of 12 h light/12 h dark) where they were maintained with a 10% sucrose solution for a maximum of 28 days. Seven *Ae*. *albopictus* populations were infected with DENV-1 (F0_SM, F0_SG, F0_SPh, F0_SA, F2_SC, F2_BP, F2_TB), three populations were infected with DENV-2_JUL (F1_SPa, F1_SL, F1_LP), and four populations with DENV-2_EVAg (F0_SM, F0_SG, F0_SPh, F0_SA) (Table 1). The *Ae*. *aegypti* colony (F_31_ and F_37_ generations) was infected with the three DENV strains (Table 1). Mosquito populations infected with a specific DENV strain were selected based on the availability of the both mosquito populations and the viral strains in the laboratory.

### Vector competence analysis

At 14, 21, and 28 days post-exposure (dpe) to infectious blood meals, legs and wings of mosquitoes (N = 5 to 76, Table 1) were removed before salivation [45]. Saliva from individual mosquitoes was collected for 30 min by inserting the proboscis into a pipette tip containing 5 μl of FBS. Afterwards, the solution contained in the tip was transferred to 45 μl of complete MEM medium (i.e. MEM supplemented with l-glutamine, sodium pyruvate, penicillin, streptomycin and fungizone as described above). Then the head and the body (thorax and abdomen) were separated and ground in 200 μl of complete MEM medium supplemented with 2% FBS. After a centrifugation at 10,000×g for 5 minutes to pellet tissue debris, 150 μl of the supernatant of each sample was stored at -80°C until detection and titration of DENV. For bodies, pellets were stored in -80°C for the measurement of *Wolbachia* density (see below). The detection of DENV in bodies, heads and saliva was performed by plaque forming unit (PFU) assays on Vero cells. For bodies and heads, 48-well culture plates were seeded with 5×10^4^ Vero E6 cells per well. For saliva, 12-well plates were seeded with 3×10^5^ Vero E6 cells per well. The following day, cells were incubated for 2 hours (37°C, 5% CO_2_) with 100 μl of ten-fold dilutions of body or head homogenates, or with 250 μl of ten-fold dilutions of the solution containing saliva. All dilutions were performed with complete MEM medium supplemented with 2% FBS. Then, 200 μl (for bodies and heads) or 1 ml (for saliva) of MEM medium supplemented with 5% of FBS and 0.8% of carboxymethylcellulose sodium salt (CMC; Sigma-Aldrich, Saint-Quentin-Fallavier, France) were added to each well. After 5 days of incubation (37°C, 5% CO_2_), supernatants were removed, cells were washed twice with PBS, fixed with 3.7% paraformaldehyde (Sigma-Aldrich), and stained with 0.5% crystal violet (Sigma-Aldrich) dissolved in ethanol 20% (S1 Fig). Vector competence of each population was evaluated based on three parameters: the infection rate (IR), the dissemination efficiency (DE), and the transmission efficiency (TE). IR, DE and TE correspond respectively to the proportion of infected bodies, head and saliva among the total number of mosquitoes tested. For each DENV strain, the number of mosquitoes analyzed per population and the selected dpe depended on the total number of females having taken an infectious blood meal.

### Viral RNA extraction and amplification

Viral loads in the bodies of infected F_0_ *Ae*. *albopictus* mosquitoes (N = 43) previously exposed to DENV-1 and collected at 21 and 28 dpe were quantified using the reverse transcription quantitative real-time PCR (RT-qPCR). These samples are sub-samples of those tested for vector competence (infection rates). RNA from mosquito bodies was individually extracted using the QIAcube HT robotic workstation and the associated Cador Pathogen 96 QIAcube HT Kit (QIAGEN) following manufacturer’s recommendations with slight modifications. Extracted RNA was eluted in 100 μl of AVE buffer (QIAGEN). The RT-qPCR was then performed using the QIAGEN OneStep RT-PCR Kit according to the manufacturer’s recommendations. For this, a mixed solution was prepared with RNA template (5 μl), a TaqMan probe (FAM-ACACCTCAAGCTAA-TAMRA), and primers (Forward 5’-GAACATGGRACAAYTGCAACYAT-3’; Reverse 5’-CCGTAGTCDGTCAGCTGTATTTC-3’) specific for the DENV-1 viral envelope gene. The thermocycler program consisted of a reverse transcription step of 45 min at 45°C, denaturation for 5 min at 95°C followed by 40 cycles of amplification (72°C for 5 s and 56°C for 60 s). The number of viral RNA copies was estimated against a standard curve following the methodology published by the HAS (Haute Autorité de Santé, France). Briefly, plasmids containing targeted DENV-1 were synthesized by GeneCust (France) and used as the standard curve at concentrations of 10^1^ to 10^8^ RNA copies per μl.

### *Wolbachia* density

*Wolbachia* were quantified in bodies of F_0_ *Ae*. *albopictus* mosquitoes (N = 75) previously exposed to DENV-1 and collected at 21 and 28 dpe. DNA was extracted from individual body carcasses, previously stored in -80°C from samples used to test vector competence (infection rates), using the QIAcube HT robotic workstation and the associated Cador Pathogen 96 QIAcube HT Kit (QIAGEN) following the manufacturer’s recommendations with slight modifications. Afterwards, DNA was eluted in 100 μl of AVE buffer (QIAGEN) and stored at -20°C until molecular investigations. Real-time quantitative PCRs were performed with the CFX96 Touch Real-Time PCR Detection System (Bio-Rad, Hercules, CA, USA) to estimate the number of *Wolbachia* genome copies in each sample. Two PCRs that specifically amplified the *Wolbachia* surface protein gene *wsp* for the strains *w*AlbA and *w*AlbB were performed using newly designed primers for *w*AlbA (*wsp*A: wspA_F 5’-TAACAGCAATTTCAGGACTAG-3’ and wspA_R 5’-CTGTTTTGATTATTTATAGCGG-3’) and *w*AlbB (*wsp*B: wspB_F 5’-GTGGCAGTATTTTCAGGATTG-3’ and wspB_R 5’-CTGCACTAGCTTCTGAAGG-3’) that amplify 140 bp and a 130 bp fragments, respectively. *Wolbachia* genomes were quantified relative to mosquito genomes. To this end, a fragment of the *Ae*. *albopictus* 40S ribosomal protein S7 (RSP7) gene of 140 bp was amplified with designed primers (RSP7_F 5’-ATCGAGTTCAACAGCAAGAA-3’ and RSP7_R 5’-CGACGTGCTTGCCGGAGAAC-3’). About 5 ng of genomic DNA was mixed with 10 μl of QuantiNova Probe RT-PCR master mix (QIAGEN), 1 μl (10 μM) of each primer and 3.6 μl of RNase-free water. PCRs were run with activation for 2 mins at 95°C followed by 45 cycles (95°C for 5s and 60°C for 5s). Each DNA template was analyzed in triplicate for *wsp*A, *wsp*B and RSP7. A standard curve was generated for each qPCR run to standardize the signals with the RSP7 reference. The relative mean genome number of *w*AlbA and *w*AlbB strains was obtained per RSP7 copy number.

### Statistical analysis

#### Vector competence for DENV

A first analysis was performed on all data using the proportion test to compare IR, DE and TE parameters between mosquito populations for each dpe and each DENV strain separately. A Bonferroni correction was applied for multiple comparisons [46]. A second analysis was performed to study the effects of four explanatory parameters on the vector competence of *Ae*. *albopictus* populations to DENV-1 using generalized linear models (GLM) with a binomial error structure (or quasi-binomial in case of over-dispersed data). The explanatory parameters tested were all categorical: ‘*population’* (seven modalities: F0_SM, F0_SA, F0_SG, F0_SPh, F2_SC, F2_BP and F2_TB), ‘*generation’* (two modalities: F_0_ and F_2_), ‘*dpe*’ the day post-exposure (three modalities: 14 dpe, 21 dpe, and 28 dpe), and ‘*area’* (four modalities: North, East, South and West). The GLM analyses were made independently for each of the three vector competence parameters (i.e. IR, DE, and TE) used as binary response variables (DENV infected and non-infected). As each population had only one generation, the effect of the two parameters ‘*population’ (of F*_*0*_ *or F*_*2*_ *generation)* and ‘*generation’* were analyzed independently to avoid any confounding effect. Therefore, three distinct models were used: GLM1 with the following maximal model *on F*_*0*_ *generation* only “population * dpe”, GLM2 with on *F*_*2*_ *generation* only “population * dpe”, and GLM3 on all populations with “(*generation* + *dpe* + *area)^2*”. Selection of the minimal model was assessed using the likelihood ratio test (LRT), and the significance of the selected parameters addressed by Anova from the *car* R package [47]. Based on the minimal model selected, the emmeans R package [48] was used to assess the statistical difference between the modalities.

#### Wolbachia density

We first compared *Wolbachia* density (*w*AlbA, *w*AlbB, or *w*AlbTot) according to dpe for each population using the Mann_Whitney test for unpaired samples and no significant difference was noted between 21 and 28 dpe either for *w*AlbA, *w*AlbB, or *w*AlbTot (Mann_Whitney tests; all p-values > 0.0789). Therefore, subsequent analyses were carried out by combining samples from both dpe (N = 75, 41 samples from 21 dpe and 34 samples from 28 dpe). The Mann_Whitney test was also used to compare the densities of the two *Wolbachia* strains for each mosquito population. The effect of population on *Wolbachia* density (*w*AlbA, *w*AlbB, or *w*AlbTot) was explored using the non-parametric Kruskal-Wallis analysis followed by pairwise post-hoc comparisons of medians with a Dunn’s test. To assess the influence of *Wolbachia* on vector competence parameters, mosquitoes were first classified according to four IDT (Infection, Dissemination, Transmission) scores (0, 1, 2 or 3). These IDT scores were defined as follows: the IDT score 0 for mosquitoes with no infectious DENV-1 particles either in the body, head or saliva; the IDT score 1 for samples with only infected bodies; the IDT score 2 for mosquitoes with infectious particles in the bodies and the heads; and the IDT score 3 for mosquitoes with infectious DENV-1 particles in the bodies, heads and saliva (S1 Table). Then *Wolbachia* density medians were compared between different combinations of these IDT scores using Mann_Whitney tests for unpaired samples. The correlation between *Wolbachia* densities and DENV-1 viral load in the bodies of infected mosquitoes was examined using a Pearson correlation coefficient test in a sub-sample of DENV-1 infected *Ae*. *albopictus* (N = 43). The effect of mosquito population on DENV-1 viral load was also explored using the non-parametric Kruskal-Wallis analysis followed by pairwise post-hoc comparisons of medians with a Dunn’s test.

All the statistical analyses were performed in R software (v.3.6.2) (R Core Team 2019) with also the following packages: PropCIs [49], stats (R Core Team 2019), ggplot2 [50], rstatix [51], ggbreak [52], and glm2 [53].

## Results

*Aedes albopictus* populations of three generations (F_0_, F_1_ and F_2_) collected in different geographic areas (North, East, South and West) in Reunion Island, and a laboratory colony of *Ae*. *aegypti* (F_31_ and F_37_ generations) used as control, were exposed to three DENV strains: one DENV-1 and two DENV-2 that have circulated on the island between 2018–2020 (Table 1).

### No transmission of DENV-2 strains by *Aedes albopictus* populations

Three *Ae*. *albopictus* populations of F_1_ generation (F1_SPa, F1_SL and F1_LP) were exposed to the DENV-2_JUL strain and vector competence parameters were examined at 14 dpe. Low IR were observed with values ranging between 5.88% (95% Confidence Interval = 1.05–26.98%) and 11.76% (3.29–34.34%) (S2 Table) but no significant differences between populations were observed in pairwise comparisons (all *p-values* > 0.99). No dissemination or transmission of DENV-2_JUL was observed for the three *Ae*. *albopictus* populations (S2 Table). In comparison, a laboratory colony of *Ae*. *aegypti* exposed to DENV-2_JUL was examined at 14 and 21 dpe. Similar to *Ae*. *albopictus*, low IRs were observed: 9.38% (3.24–24.22%) and 8.82% (3.05–22.96%) at 14 and 21 dpe, respectively, and no dissemination or transmission of the DENV-2_JUL strain at either dpe (S2 Table).

Four *Ae*. *albopictus* populations of F_0_ generation (F0_SM, F0_SA, F0_SG, and F0_SPh) were exposed to the DENV-2_EVAg strain and vector competence parameters were examined at 14, 21, and 28 dpe for F0_SM and F0_SA, and only at 28 dpe for F0_SG and F0_SPh. The *Ae*. *albopictus* populations also showed very low vector competence to DENV-2_EVAg. In F0_SM and F0_SA populations, only one infected body was observed at 14 dpe (IR = 2.08%; 0.37–10.90%; for both populations), and no infection was observed later at 21 or 28 dpe (S3 Table). At 28 dpe, no infected body was observed in the F0_SG population (S3 Table), while IR in the F0_SPh population was estimated at 25% (10.18–49.50%), and was significantly higher than in F0_SM and F0_SA population (IR = 0.00%; 0.00–7.41% for both populations; and *p-values =* 0.009 for both comparisons). No dissemination or transmission was observed for the different populations and dpe considered (S3 Table). For *Ae*. *aegypti*, IR was 8.57% (2.96–22.38%) at 14 dpe, and, unlike *Ae*. *albopictus* populations, virus dissemination was observed (DE = 2.86%; 0.51–14.53%) for the DENV-2_EVAg strain. However, as with the *Ae*. *albopictus* populations, no transmission was observed at 14 dpe for *Ae*. *aegypti* (S3 Table).

### Transmission of the DENV-1 strain by *Aedes albopictus* populations

**Fig 2:**
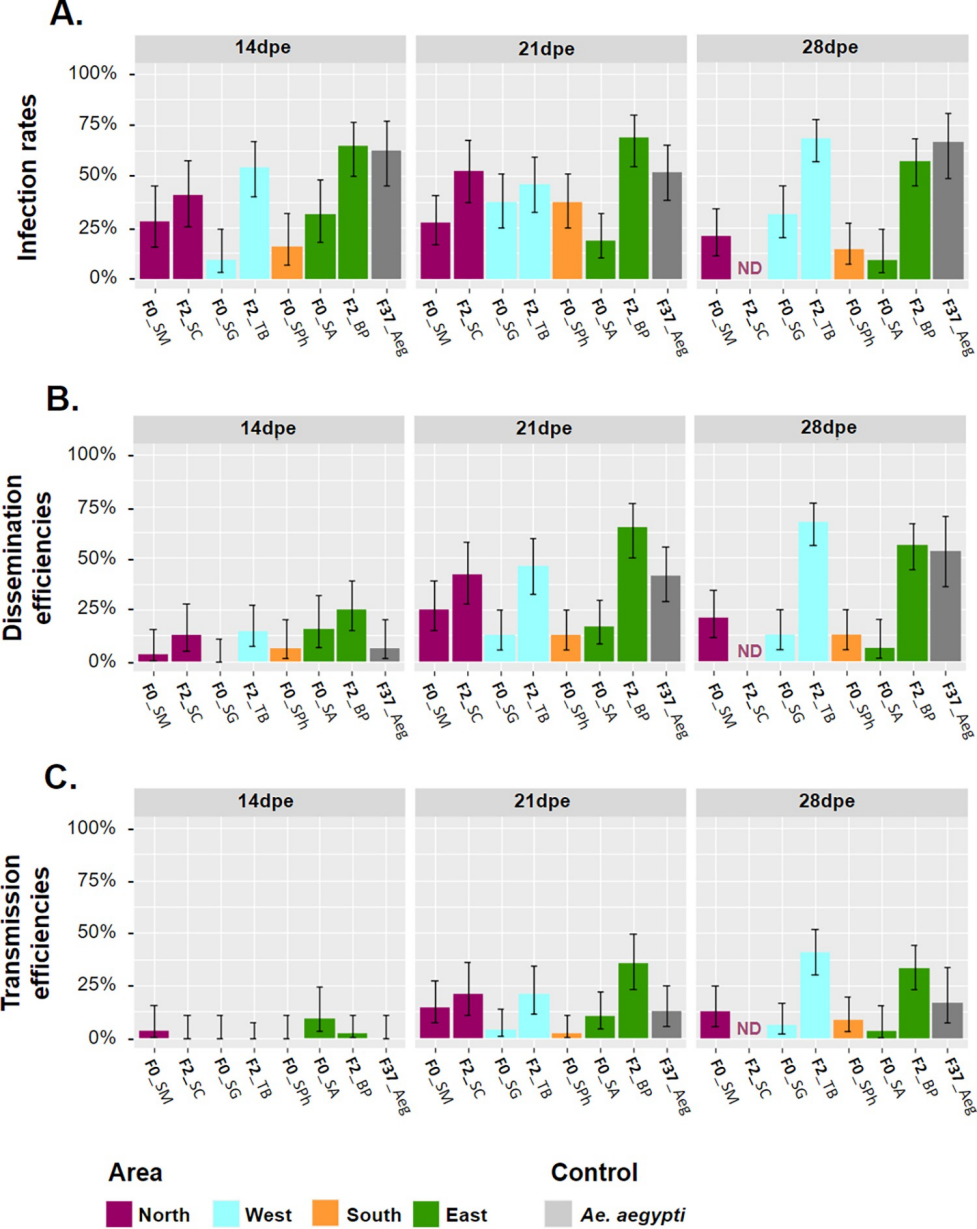
Vector competence parameters of *Aedes albopictus* and *Aedes aegypti* populations from Reunion Island exposed to the DENV-1 strain. **(A.)** Infection rates (IR), **(B.)** dissemination efficiencies (DE), and **(C.)** transmission efficiencies (TE) of mosquito populations according to geographic areas (orange: South; blue: West; purple: North; and green: East). Data obtained with *Ae*. *aegypti* are shown in gray. Vector competence parameters were examined at 14, 21, and 28 days post-exposure (dpe) to the DENV-1 strain via an infectious blood meal. Error bars correspond to the 95% confidence intervals. ND = not determined.

We examined the vector competence of four *Ae*. *albopictus* populations of F_0_ generation (F0_SM, F0_SA, F0_SG and F0_SPh) and three populations of F_2_ generation (F2_SC, F2_BP and F2_TB), as well as a laboratory colony of *Ae*. *aegypti*, at 14, 21, and 28 dpe after exposure to the DENV-1 strain (Fig 2 and Table 2).

**Table 2:** Vector competence parameters of *Aedes albopictus* and *Aedes aegypti* populations exposed to the DENV-1 strain. Infection rates (IR), dissemination efficiencies (DE), and transmission efficiencies (TE) were examined at 14, 21, and 28 days post-exposure (dpe) to an infectious blood meal. IR = number of infected bodies among the mosquitoes tested (%); DE = number of infected heads among the mosquitoes tested (%); TE = number of infected saliva among the mosquitoes tested (%). The numbers in brackets correspond to the 95% confidence interval, while the numbers in parentheses represent the count of positive samples out of the total samples tested. ND = not done. F0_SM, F0_SA, F0_SG, F0_SPh, F2_SC, F2_BP and F2_TB correspond to *Ae*. *albopictus* populations and F37_Aeg is the *Ae*. *aegypti* population.

Population	14 dpe			21 dpe			28 dpe		
	IR	DE	TE	IR	DE	TE	IR	DE	TE
**F0_SM**	28.13% [15.56–45.37%] (9/32)	3.13% [0.55–15.74%] (1/32)	3.13% [0.55–15.74%] (1/32)	27.08% [16.57–41.00%] (13/48)	25.00% [14.92–38.78%] (12/48)	14.58% [7.25–27.17%] (7/48)	20.83% [11.73–34.26%] (10/48)	20.83% [11.73–34.26%] (10/48)	12.50% [5.86–24.70%] (6/48)
**F0_SA**	31.25% [17.95–48.57%] (10/32)	15.63% [6.86–31.75%] (5/32)	9.38% [3.24–24.22%] (3/32)	18.75% [10.19–31.94%] (9/48)	16.67% [8.70–29.58%] (8/48)	10.42% [4.53–22.17%] (5/48)	9.38% [3.24–24.22%] (3/32)	6.25% [1.73–20.15%] (2/32)	3.13% [0.55–15.74%] (1/32)
**F0_SG**	9.38% [3.24–24.22%] (3/32)	0.00% [0.00–10.72%] (0/32)	0.00% [0.00–10.72%] (0/32)	37.50% [25.22–51.64%] (18/48)	12.50% [5.86–24.70%] (6/48)	4.17% [1.15–13.98%] (2/48)	31.25% [19.95–45.33%] (15/48)	12.50% [5.86–24.70%] (6/48)	6.25% [2.15–16.84%] (3/48)
**F0_SPh**	15.63% [6.86–31.75%] (5/32)	6.25% [1.73–20.15%] (2/32)	0.00% [0.00–10.72%] (0/32)	37.50% [25.22–51.64%] (18/48)	12.50% [5.86–24.70%] (6/48)	2.08% [0.37–10.90%](1/48)	14.58% [7.25–27.17%] (7/48)	12.50% [5.86–24.70%] (6/48)	8.33% [3.29–19.55%] (4/48)
**F2_SC**	40.63% [25.52–57.74%] (13/32)	12.50% [4.97–28.07%] (4/32)	0.00% [0.00–10.72%] (0/32)	52.63% [37.26–67.52%] (20/38)	42.11% [27.85–57.81%] (16/38)	21.05% [11.07–36.35%] (8/38)	ND	ND	ND
**F2_BP**	64.58% [50.44–76.57%] (31/48)	25.00% [14.92–38.78%] (12/48)	2.08% [0.37–10.90%] (1/48)	68.75% [54.67–80.05%] (33/48)	64.58% [50.44–76.57%] (31/48)	35.42% [23.43–49.56%] (17/48)	57.14% [45.48–68.06%] (40/70)	55.71% [44.08–66.75%] (39/70)	32.86% [23.00–44.50%] (23/70)
**F2_TB**	54.17% [40.29–67.42%] (26/48)	14.58% [7.25–27.17%] (7/48)	0.00% [0.00–7.41%] (0/48)	45.83% [32.58–59.71%] (22/48)	45.83% [32.58–59.71%] (22/48)	20.83% [11.73–34.26%] (10/48)	68.42% [57.30–77.77%] (52/76)	67.11% [55.94–76.62%] (51/76)	40.79% [30.44–52.02%] (31/76)
**F37_Aeg**	62.50% [45.25–77.07%] (20/32)	6.25% [1.73–20.15%] (2/32)	0.00% [0.00–10.72%] (0/32)	52.08% [38.33–65.53%] (25/48)	41.67% [28.85–55.72%] (20/48)	12.50% [5.86–24.70%] (6/48)	66.67% [48.78–80.77%] (20/30)	53.33% [36.14–69.77%] (16/30)	16.67% [7.34–33.56%] (5/30)

All populations were susceptible to DENV-1 infection (Fig 2A and Table 2): IRs values ranged from 9.38% (3.24–24.22%) to 64.58% (50.44–76.57%) at 14 dpe, from 18.75% (10.19–31.94%) to 68.75% (54.67–80.05%) at 21 dpe, and from 9.38% (3.24–24.22%) to 68.42% (57.30–77.77%) at 28 dpe (Fig 2A and Table 2). Statistical analysis of IR showed that the explanatory parameters "dpe", and "area" were not retained in the minimal models of the three GLMs, and therefore had no effect on the IR values (Table 3). Only the "generation" parameter showed a significant influence on IR (GLM3, X^2^ = 51.024, df = 1, *p-value* < 0.0001), with a higher IR for populations of F_2_ generation (IR = 58.09%; 53.25–62.78%) than for populations of F_0_ generation (IR = 24.19%; 20.63–28.15%; *p-value* < 0.0001, S4 Table) but no significant difference was noted between populations of the same generation (all *p-values* > 0.05).

**Table 3:** Statistical analyses of vector competence parameters of *Ae*. *albopictus* populations infected with the DENV-1 strain. Mosquitoes were examined at 14, 21 and 28 days post-exposure (dpe). In these analyses, the influence of mosquito population, dpe, generation and area were tested. d.f. is the degree of freedom and *X***^2^** is the Chi-square value.

	Maximal model	Parameters retained in the minimal model	Generation	IR			DE			TE		
				*X^2^*	d.f.	p-value	*X^2^*	d.f.	p-value	*X^2^*	d.f.	p-value
***GLM1***	*population of F*_*0*_ *generation + dpe*	***population***	***F0***	-	-	-	-	-	-	-	-	-
		***dpe***		-	-	-	-	-	-	-	-	-
***GLM2***	*population of F*_*2*_ *generation + dpe*	***population***	***F2***	-	-	-	-	-	-	-	-	-
		***dpe***		-	-	-	30.485	2	<0.0001	60.324	2	0.0001
***GLM3***	*(generation + dpe + area)^2*	***generation x dpe***	***All***	-	-	-	*-*	-	-	154.34	2	<0.0001
		***generation x area***		-	-	-	-	-	-	38.09	2	<0.0001
		***dpe x area***		-	-	-	-	-	-	185.61	6	<0.0001
		***generation***		51.024	1	<0.0001	61.320	1	<0.0001	461.31	1	<0.0001
		***dpe***		-	-	-	29.334	2	<0.0001	811.59	2	<0.0001
		***area***		-	-	-	-	-	-	47.41	3	<0.0001

All *Ae*. *albopictus* populations, as well as the *Ae*. *aegypti* colony, were able to disseminate DENV-1 (Fig 2B and Table 2). Similar to IR, pairwise comparisons revealed significantly higher DE for populations of F_2_ generation compared to those of F_0_ generation, but only at 21 and 28 dpe (S4 Table), and no significant difference was noted between populations of the same generation (all *p-values P* > 0.05). Analyzing both generations separately, DE were not different between populations ("population" parameters not retained in the GLM1 and GLM2 minimal models; Table 3), whereas the "generation" parameter showed a significant influence (GLM3, *X^2^* = 61.320, df = 1, *p-value* < 0.0001) with higher mean DE for populations of F_2_ generation (DE = 44.61%; 39.86–49.46%) than for populations of F_0_ generation (DE = 12.90%; 10.24–16.14%; *p-value* < 0.0001). In contrast to IR, statistical analyses showed a significant effect of dpe, with DE increasing over time for populations of F_2_ generation (GLM2, *X^2^* = 30.485, df = 2, *p-value* < 0. 0001) from 17.97% (12.28–25.52%) at 14 dpe to 51.49% (43.11–59.79%) at 21 dpe or to 61.64% (53.55–69.14%) at 28 dpe (pairwise comparison, *p-value* = 0.0003 and *p-value* < 0.0001, respectively). For populations of F_0_ generation, no effect of "dpe" was observed (GLM1, Table 3), with mean DEs ranging from 3.13% (0.55–15.74%) to 15.63% (6.86–31.75%) at 14 dpe, from 12.50% (5.86–24.70%) to 25.00% (14.92–38.78%) at 21 dpe, and from 6.25% (1.73–20.15%) to 20.83% (11.73–34.26%) at 28 dpe (Table 2). When both generations are analyzed together, “dpe” significantly influenced DE (GLM3, *X^2^* = 29.334, df = 2, *p-value* < 0.0001) with DE significantly increasing between 14 dpe (DE = 12.11%; 8.66–16.68%) and 21 dpe (DE = 30.98%; 26.21–36.20%) or 28 dpe (DE = 35.40%; 30.38–40.77%) (pairwise comparison, *p-value* = 0.0001 and *p-value* < 0.0001, respectively). Finally, and as for the IR, the “area” parameter was not retained in the GLM3 minimal model, demonstrating no influence on the DE values (Table 3).

The *Ae*. *albopictus* populations and the *Ae*. *aegypti* colony tested were all able to transmit the DENV-1 strain tested at 21 dpe, with F0_SM, F0_SA and F2_BP populations containing infectious virus particles in the saliva as soon as 14 dpe (Fig 2C and Table 2). As with the DE, significantly higher TE was found for populations of F_2_ generation compared to populations of F_0_ generation at both 21 and 28 dpe (S4 Table), but no significant difference was noted between populations of the same generation (all *p-values* > 0.05). Only the “dpe” parameter was retained in the GLM2 minimal model (GLM2, *X^2^* = 60.324, df = 2, *p-value* < 0.0001), with a significantly lower TE for the populations of F_2_ generation at 14 dpe (TE = 0.78%; 0.14–4.29%) than at 21 dpe (26.12%; 19.42–34.15%, *p-value* = 0.002) or than at 28 dpe (36.99%; 29.58–45.06%, *p-value =* 0.0003). TE were not significantly different between the populations of F_0_ generation (GLM1) and ranged from 3.13% (0.55–15.74%) to 9.38% (3.24–24.22%) at 14 dpe, from 2.08% (0.37–10.90%) to 14.58% (7.25–27.17%) at 21 dpe, and from 3.13% (0.55–15.74%) to 12.50% (5.86–24.70%) at 28 dpe (Fig 2C and Table 2). For the GLM3, all the explanatory parameters and their two-by-two interactions were retained in the minimal model (Table 3). Post-hoc analyses showed differences in TE between generations within certain areas. In the West, the population of F_2_ generation had a significantly higher TE (TE = 23.84%; 18.09–30.73%) than that of F_0_ generation (TE = 3.91%; 1.68–8.82%; *p-value* < 0.0001). In the East, the population of F_2_ generation had a significantly higher TE (TE = 25.90%; 19.84–33.06%) than that of F_0_ generation (TE = 8.04%; 4.29–14.57%; *p-value* < 0.0001). Moreover, TE increased over time according to the area with, for example, a mean TE for the northern populations increasing significantly between 14 dpe (TE = 1.56%; 0.28–8.33%) and 21 dpe (TE = 17.44%; 10.86–26.80%; *p-value* < 0.0001) and between 14 and 28 dpe (TE = 12.50%; 5.86–24.70%; *p-value* < 0.0001). The *Ae*. *aegypti* colony presented similar TEs to *Ae*. *albopictus* with 12.50% (5.86–24.70%) and 16.67% (7.34–33.56%) at 21 and 28 dpe, respectively (Fig 2C and Table 2).

We also tested the influence of the sampling period on vector competence to the DENV-1 strain by comparing IRs, DEs and TEs at 14, 21 and 28 dpe between two populations collected in the same location (F0_SM) a month apart. No significant difference was observed between the two populations for either IRs (*p-values* > 0.760, for all the dpe tested), DEs (*p-values* > 0.356), or TEs (*p-values* > 0.425) (see S5 Table for all proportion data).

### No effect of *Wolbachia* on the replication of DENV-1 in *Aedes albopictus*

We measured the density of *Wolbachia* strains *w*AlbA, *w*AlbB and of both strains (*w*AlbTot, i.e. *w*AlbA + *w*AlbB) in individual *Ae*. *albopictus* mosquitoes (N = 75) from the four populations of F_0_ generation (F0_SM, F0_SG, F0_SPh, F0_SA) previously exposed to DENV-1 infectious blood meals and collected at 21 and 28 dpe. These two dpe were selected because they showed higher values of vector competence parameters in particular DE and TE (see Fig 2), thus allowing testing the correlation between *Wolbachia* densities and vector competence referred as IDT scores (S1 Table). The *w*AlbA strain presented a significantly higher density than the *w*AlbB strain in the four populations (Mann_Whitney tests; *p-value* = 0.002 for F0_SM and for F0_SA; *p-value* < 0.001 for F0_SG and for F0_SPh), with median densities per population ranging from 2.20 (0.70–2.20) to 9.00 (3.90–24.80) bacteria/cell for *w*AlbA and from 0.40 (0.23–0.60) to 1.95 (1.40–2.30) bacteria/cell for *w*AlbB (S6 Table). The density of both *Wolbachia* strains varied according to mosquito populations (S6 Table), with the lowest densities observed in the F0_SG population, followed by the F0_SPh population and higher densities in the F0_SM and F0_SA populations (S2 Fig and S6 Table).

To examine the influence of *Wolbachia* density on *Ae*. *albopictus* vector competence, the samples from the four populations and from both 21 and 28 dpe were gathered (since no significant difference was found between dpe and between populations for F_0_ generation, i.e. GLM1, see Table 3). They were then classified according to their IDT score (N = 12 to 19 mosquitoes per IDT score, S7 Table). No significant difference in *Wolbachia* density was observed between the IDT score 0 (i.e. no infection) and all three other IDT groups (1, 2 and 3) gathered (i.e. with at least one infected tissue) either for *w*AlbA (Mann_Whitney test, *p-value* = 0.609), *w*AlbB (*p-value* = 0.613), or *w*AlbTot (*p-value* = 0.696), suggesting that *Wolbachia* density did not affect the ability of mosquitoes to become infected after exposure to DENV-1 (S3 Fig, S7 Table). Similarly, no significant difference was found by comparing the *Wolbachia* density between the score IDT 1 on one hand, and the scores 2 and 3 gathered on the other hand (S7 Table) (Mann_Whitney tests; *p-value* = 0.434 for *w*AlbA; *p-value* = 0.066 for *w*AlbB; *p-value* = 0.494 for *w*AlbTot), suggesting that *Wolbachia* density did not affect the ability of mosquitoes to disseminate the DENV-1 after being infected (S3 Fig). *Wolbachia* density had also no impact on the ability of mosquitoes to transmit DENV-1 after dissemination in the heads, a result highlighted by the absence of any significant difference between the *Wolbachia* density of IDT scores 2 and 3 (S7 Table) (Mann_Whitney tests; *p-value* = 0.151 for *w*AlbA; *p-value* = 0.238 for *w*AlbB; *p-value* = 0.113 for *w*AlbTot) (S3 Fig).

**Fig 3:**
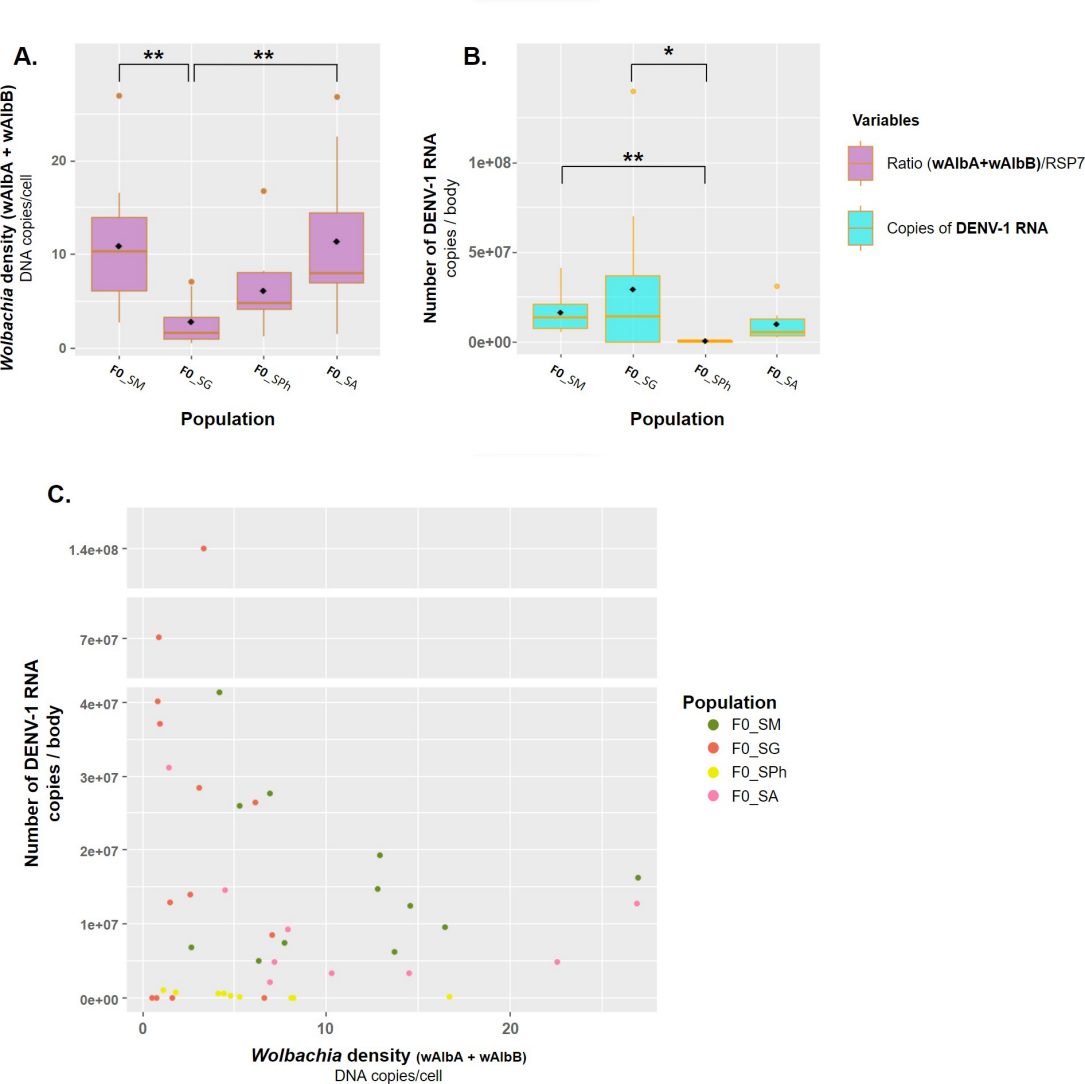
Correlation between *Wolbachia* density and viral load of the DENV-1 strain in *Aedes albopictus* mosquitoes from Reunion Island. The mosquitoes tested belong to the populations of F_0_ generation from Sainte-Marie (F0_SM), Saint-Gilles les Hauts (F0_SG), Saint-Philippe (F0_SPh), or Saint-André (F0_SA), and were collected 21 or 28 days post-exposure (dpe) to the DENV-1 local strain. **(A.)** The total *Wolbachia* density (*w*AlbTot *i*.*e*. *w*AlbA + *w*AlbB) is given based on the ratio between the *Wolbachia* genomes (*w*AlbA and *w*AlbB) and *Ae*. *albopictus* genomes (RSP7 concentrations) in the bodies of mosquitoes according to their population of origin. **(B.)** Number of DENV-1 RNA copies in the body of the mosquitoes according to their population of origin. In graphs A and B, the line inside each boxplot represents the median and the diamond corresponds to the mean of each population. **(C.)** Number of DENV-1 RNA copies according to the total *Wolbachia* density in the body of each mosquito tested (N = 43). *p < 0.05, **p < 0.01, Dunn’s tests.

Finally, we examined the correlation between *Wolbachia* density (*w*AlbTot i.e. *w*AlbA+*w*AlbB) and the number of DENV-1 RNA copies in the bodies of the infected mosquitoes (N = 43 positive samples for DENV-1). The median DENV-1 RNA copies per body in the examined populations ranged from 2.72×10^5^ (5.05×10^4^−7.71×10^5^) to 1.40×10^7^ (3.41×10^1^–4.01×10^7^) (S8 Table). Firstly, we compared the *Wolbachia* density (Fig 3A) or the DENV-1 viral load (Fig 3B) between the four populations. The mosquito population showed a significant effect on the median *Wolbachia* density (Kruskal-Wallis test, *X^2^* = 16.86, d.f. = 3, *p-value* < 0.001) and on the median number of DENV-1 RNA copies (Kruskal-Wallis test, *X^2^* = 13.48, d.f. = 3, *p-value* = 0.004). Although both *Wolbachia* density and DENV-1 viral load vary significantly according to mosquito populations (Fig 3A and 3B), no significant correlation between the two parameters was noted (Pearson correlation coefficient test, cor = -0.190, 95% CI = [-0.464;0.117], t = -1.236, d.f. = 41, *p-value* = 0.224) (Fig 3C), suggesting a lack of association between *Wolbachia* density and DENV-1 viral load.

## Discussion

In this study, we examined the vector competence of *Ae*. *albopictus* populations from Reunion Island exposed to local epidemic DENV-1 and DENV-2 strains that had been previously isolated and genetically characterized [43,44]. The mosquito populations used for experimental infections were collected in different geographic areas across Reunion Island and were of F_0_, F_1_ and F_2_ generations. The use of F_0_ populations allowed experimental conditions to be as close as possible to those of natural mosquito populations. We also assessed the vector competence of a laboratory colony of *Ae*. *aegypti* as control because this species is considered as the primary DENV vector [5,7,54–58]. In general, no significant difference between vector competence of *Ae*. *albopictus* populations and the *Ae*. *aegypti* colony was observed, in agreement with the results of Florida mosquito populations exposed to a DENV-1 strain [59], or of China vector populations exposed with DENV-2 strains [60].

Contrasting results were observed when *Ae*. *albopictus* populations were exposed to DENV-1 and DENV-2 strains. None of the tested populations was able to transmit the DENV-2 strains, while the DENV-1 strain was transmitted by all populations with TEs reaching 40.8% (in the F2_TB population at 28 dpe). Our results are different from those observed with *Ae*. *albopictus* populations from other geographic regions which generally show higher levels of transmission with DENV-2 strains compared to DENV-1 strains [61]. For instance, higher viral loads were described in saliva of an *Ae*. *albopictus* population from Vietnam exposed to DENV-2 compared to specimens infected with DENV-1 [58]. The results observed in *Ae*. *aegypti* are quite similar to those described in *Ae*. *albopictus* with no transmission of DENV-2 strains, while TEs of 12.5% and 16.7% were observed with DENV-1 at 21 and 28 dpe, respectively (the means TEs for *Ae*. *albopictus* being 15.5% and 17.3% at 21 and 28 dpe, respectively). The observed difference in the transmission of the two DENV serotypes by *Ae*. *albopictus* and *Ae*. *aegypti* mosquitoes could be explained by two non-exclusive hypotheses. Firstly, the viral titres in infectious blood meals were higher for the DENV-1 strain (7×10^6^ PFU/ml) than for the DENV-2 strains (between 6.8×10^4^ PFU/ml and 3.2×10^5^ PFU/ml), and viral titres in blood meals are known as a factor affecting the vector competence of mosquitoes [58,62–64]. Secondly, the difference in vector competence between DENV-1 and DENV-2 strains could be linked to a greater replicative fitness or affinity of the DENV-1 local strain with the *Ae*. *albopictus* and *Ae*. *aegypti* from Reunion Island compared to the two DENV-2 strains. It is recognized that vector competence for a given virus is the result of interactions between a viral strain, a mosquito population and a given environment [41,56,60,63,65–67]. For example, the substitution of E1-226A by E1-226V in the E1 structural protein of the Chikungunya virus has been associated with increased virus replication and transmission in *Ae*. *albopictus*, contributing to the unprecedented Chikungunya epidemic in Reunion island and other islands in South-Western Indian Ocean in 2005–2006 [68,69]. The hypothesis of a better replicative fitness is reinforced by a previous investigation with Colombian DENV strains showing a greater replicative fitness of DENV-1 compared to DENV-2 in the human hepatocyte cell line (Huh-7) [70]. It would be interesting in future investigations to compare replicative fitness of DENV-1 and DENV-2 strains from Reunion Island in human cell lines, *Ae*. *albopictus* cell lines as well as in *Ae*. *albopictus* populations using identical viral titres for both DENV serotypes.

Among the four explanatory parameters including “population”, “dpe”, “generation”, and “area” that can affect the three vector competence parameters (IR, DE, and TE), three parameters (“dpe”, “generation”, and “area”) showed a significant effect. Concerning the “dpe”, a global increase of IRs, DEs and TEs occurred over time, higher values being observed in older mosquitoes in particular at 28 dpe compared to 21 and 14 dpe. This result reflects the kinetics of replication of arboviruses inside mosquitoes, from the initial midgut infection to the release of infectious viral particles in saliva following a dissemination phase in all the tissues [71]. The other significant parameter was the “generation”, with higher vector competence parameters observed in mosquito populations of F_2_ generation than that of F_0_ generation. The effect of generation on vector competence could be explained by a reduced genetic diversity in mosquitoes reared in the laboratory due to a founder effect. The rearing of mosquitoes in the laboratory for two generations could alter the mosquito genetic diversity as well as their microbiota [63,72–75]. Indeed, mosquito microbiota, in particular midgut bacteria, have been shown to modulate vector competence in several mosquito species [57,63,76–81] including *Ae*. *albopictus* [82]. This result shows the importance of working with mosquito populations of F_0_ generation to better understand the transmission patterns of arboviruses in the field. It will be interesting in future studies to examine the evolution of vector competence, genetic diversity and microbiota in mosquitoes from the same populations across several generations. Finally, as the geographic distribution of dengue cases is often heterogeneous across the island, with the western and the southern parts being the most affected compared to the northern, eastern and central parts [22], mosquito populations used in this study were collected in areas of high (West and South) and low (North and East) DENV transmission. Our data indicated a significant influence of the area of collection on TEs alone, but also in interaction either with the “dpe” parameter or with the “generation” parameter. However, the number of mosquito populations from each area was too low (between one and three populations) to conclude whether the geographic origin of mosquitoes affects the vector competence.

We examined whether the density of the *Wolbachia* strains *w*AlbA and *w*AlbB, naturally occurring in the *Ae*. *albopictus* from Reunion Island, affect vector competence and DENV-1 replication inside the mosquitoes, since *Wolbachia* have been shown to affect the replication and transmission of pathogens such as DENV, Chikungunya virus, or Zika virus in mosquito vectors [83–89]. For all examined populations, the density of the strain *w*AlbA was higher than that of the strain *w*AlbB in accordance with results described in populations from Thailand [90] and Madagascar [91]. However, other studies have reported a higher density of *w*AlbB strain than *w*AlbA in *Ae*. *albopictus* populations from China [41,92], Taiwan [40], Greece and Corsica [93], and Thailand [90]. We observed variations in *Wolbachia* density between populations, the F0_SG population (from the West) exhibited the lowest *Wolbachia* density, followed by the F0_SPh population (from the South), the F0_SM population (from the North), and the F0_SA population (from the East) with the highest *Wolbachia* density. We also described a variation in the quantity of DENV-1 viral RNA copies in the bodies of infected mosquitoes according to their population of origin. However, no significant correlation was observed between *Wolbachia* density and DENV-1 viral load, and no difference in *Wolbachia* density was found between IDT scores, suggesting that *Wolbachia* did not influence vector competence parameters (i.e. infection, dissemination or transmission) of *Ae*. *albopictus* populations exposed to DENV-1. Although it has been previously shown that the *Wolbachia* strains infecting *Ae*. *albopictus* can affect the vector competence towards DENV [35,40,41], this interference could depend on the mosquito populations and DENV strains.

## Conclusion

Our study demonstrates that *Ae*. *albopictus* populations from Reunion Island are experimentally competent for transmitting the local DENV-1 epidemic strain, but not the DENV-2 strains. However, the low viral titres of DENV-2 used in the experiments suggest that these results should be interpreted with caution. We observed a significant effect of generation on vector competence parameters, with F_0_ generation exhibiting significantly lower infection rates, dissemination efficiencies and transmission efficiencies compared to F_2_ generation mosquitoes. No significant correlation was found between *Wolbachia* density and either vector competence parameters or viral loads of DENV-1 in infected *Ae*. *albopictus* mosquitoes. Taken together, our findings highlight the importance of using natural mosquito populations and considering various parameters for enhance the understanding of DENV transmission by mosquito vectors in the field.

## Supporting information

S1 Table Number of mosquitoes tested in each vector competence parameter to examine IDT scores. The IDT (Infection, Dissemination, Transmission) score (0, 1, 2 or 3) was defined as follows: the IDT score 0 for mosquitoes with no infectious DENV-1 particles either in the body, head or saliva; the IDT score 1 for samples with only infected bodies; the IDT score 2 for mosquitoes with infectious particles in the bodies and the heads; and the IDT score 3 for mosquitoes with infectious DENV-1 particles in the bodies, heads and saliva. These categories were defined using mosquitoes exposed to DENV-1 and collected 21 and 28 dpe. Mosquitoes were from four populations: F0_SM (Sainte-Marie), F0_SG (Saint-Gilles les Hauts), F0_SP (Saint-Philippe) and F0_SA (Saint-André).
(DOC)

S2 Table Vector competence parameters of *Aedes albopictus* and *Aedes aegypti* populations exposed to the DENV-2_JUL strain. Infection rates (IR), dissemination efficiencies (DE), and transmission efficiencies (TE) were examined at 14 and 21 days post-exposure (dpe) to an infectious blood meal. IR = number of infected bodies among the mosquitoes tested (%); DE = number of infected heads among the mosquitoes tested (%); TE = number of infected saliva among the mosquitoes tested (%). The numbers in brackets correspond to the 95% confidence interval, and the numbers in parentheses represent the number of positive samples out of the total number of samples tested. ND = not done. F1_SPa, F1_SL and F1_LP correspond to *Ae*. *albopictus* populations and F31_Aeg is the *Ae*. *aegypti* population.
(DOC)

S3 Table Vector competence parameters of *Aedes albopictus* and *Aedes aegypti* populations exposed to the DENV-2_EVAg strain. Infection rates (IR), dissemination efficiencies (DE), and transmission efficiencies (TE) were examined at 14, 21, and 28 days post-exposure (dpe) to an infectious blood meal. IR = number of infected bodies among the mosquitoes tested (%); DE = number of infected heads among the mosquitoes tested (%); TE = number of infected saliva among the mosquitoes tested (%). The numbers in brackets correspond to the 95% confidence interval, and the numbers in parentheses represent the number of positive samples out of the total number of samples tested. ND = not done. F0_SM, F0_SA, F0_SG and F0_SPh correspond to *Ae*. *albopictus* populations and F31_Aeg is the *Ae*. *aegypti* population.
(DOC)

S4 Table Pairwise proportion tests between the vector competence parameters of *Aedes albopictus* populations after exposure to the DENV-1 strain. All dpe were tested independently. The comparison of the vector competence parameters (IR, DE, or TE) were performed with pairwise proportion comparison tests for each parameter and each dpe independently. The numbers in brackets correspond to the 95% confidence interval, and the numbers in parentheses represent the number of positive samples out of the total number of samples tested. Only comparisons with a significant difference (*P* < 0.05) are presented in the table.
(DOC)

S5 Table Vector competence parameters of two *Aedes albopictus* populations collected in the same locality (Sainte-Marie) at two different times of the year and exposed to the DENV-1 strain. Infection rates (IR), dissemination efficiencies (DE), and transmission efficiencies (TE) were examined at 14, 21, and 28 days post-exposure (dpe) to an infectious blood meal. IR = number of infected bodies among the mosquitoes tested (%); DE = number of infected heads among the mosquitoes tested (%); TE = number of infected saliva among the mosquitoes tested (%). The numbers in brackets correspond to the 95% confidence interval, and the numbers in parentheses represent the number of positive samples out of the total number of samples tested. ND = not done. F0_SM and F0_SM-bis correspond to *Ae*. *albopictus* populations of Sainte-Marie collected on the field in April and May 2021, respectively.
(DOC)

S6 Table Densities of *Wolbachia w*AlbA and *w*AlbB strains in the bodies of *Aedes albopictus* mosquitoes from different populations after exposure to DENV-1 strain. The mosquitoes of F_0_ generation, belonging to the populations of Sainte-Marie (F0_SM), Saint-Gilles les Hauts (F0_SG), Saint-Philippe (F0_SPh) and Saint-André (F0_SA), were examined at 21 and 28 days after being exposed to infectious blood meals containing the DENV-1 strain. In this table N = number of mosquitoes tested; 95% CI, 95% confidence interval. *w*AlbTot = *w*AlbA + *w*AlbB.
(DOC)

S7 Table Densities of *Wolbachia w*AlbA and *w*AlbB strains in the bodies of *Aedes albopictus* mosquitoes according to IDT scores. The mosquitoes of F_0_ generation, belonging to the populations of Sainte-Marie (F0_SM), Saint-Gilles les Hauts (F0_SG), Saint-Philippe (F0_SPh) and Saint-André (F0_SA) and examined at 21 and 28 days after being exposed to infectious blood meals containing the DENV-1 strain, were pooled according to their IDT scores (0, 1, 2 or 3). IDT scores are defined as follows: IDT score 0 for mosquitoes with no infectious DENV-1 particles either in the body, head or saliva; the IDT score 1 for samples with only infected bodies; the IDT score 2 for mosquitoes with infectious particles in the bodies and the heads; and the IDT score 3 for mosquitoes with infectious DENV-1 particles in the bodies, heads and saliva. In this table: N, number of mosquitoes tested; 95% CI, 95% confidence interval.
(DOC)

S8 Table The number of DENV-1 RNA copies in the bodies of mosquitoes from Reunion Island previously exposed to a DENV-1 local strain. The mosquitoes of F_0_ generation, belonging to the populations of Sainte-Marie (F0_SM), Saint-Gilles les Hauts (F0_SG), Saint-Philippe (F0_SPh), or Saint-André (F0_SA) and examined at 21 and 28 days post-exposure (dpe) to infectious blood meals containing the DENV-1 local strain. In this table: N, number of mosquitoes tested; sd, standard deviation; 95% CI, 95% confidence interval; med = median.
(DOC)

S1 Fig Examples of Plaque-Forming Units (PFU) assays of mosquito samples (A, bodies and heads; B, saliva) from the F0_SM population infected with the DENV-1 strain. For bodies and heads, tenfold serial dilutions (-1 to -3) from each specimen were performed. Monolayers of Vero cells in 48 -well plates (for bodies and heards) or 12_well plates (for saliva) were infected, incubated for 2 h at 37 o C with 5% CO2 incubator. After 5 days of incubation, plates were fixed and stained with crystal violet. Number 1, 2, 5, 9, 24, 35 and 37 correspond to samples. Number corresponding to positive saliva (5, 9 and 24) are in yellow.
(DOC)

S2 Fig Densities of *Wolbachia w*AlbA and *w*AlbB strains in the bodies in *Aedes albopictus* mosquitoes from Reunion Island. The mosquitoes of F_0_ generation from four populations (N = 75) namely Sainte-Marie (F0_SM), Saint-Gilles les Hauts (F0_SG), Saint-Philippe (F0_SPh) and Saint-André (F0_SA) were tested for their *Wolbachia* densities after being exposed to infectious blood meals containing the DENV-1 strain. For each sample, the value provided corresponds to the mean of a triplicate measure. The densities of *Wolbachia w*AlbA (orange), or *w*AlbB (green), or *w*AlbTot (*w*AlbA + *w*AlbB) (violet), are given based on the ratio between the *Wolbachia* and RSP7 concentrations which provided the number of *Wolbachia* genomes relative to the *Ae*. *albopictus* genomes. dpe = days post-exposure.
(DOC)

S3 Fig Densities of *Wolbachia w*AlbA and *w*AlbB strains in the bodies of *Aedes albopictus* mosquitoes according to vector competence parameters represented by IDT scores. These analyses were performed using individual mosquito of F_0_ generation, belonging to the populations of Sainte-Marie (F0_SM), Saint-Gilles les Hauts (F0_SG), Saint-Philippe (F0_SPh), or Saint-André (F0_SA) and examined at 21 and 28 days after being exposed to infectious blood meals containing the DENV-1 strain. The densities of *Wolbachia w*AlbA (orange), or *w*AlbB (green), or *w*AlbTot (*w*AlbA + *w*AlbB) (violet) (violet) are given based on the ratio between the *Wolbachia* and RSP7 concentrations which provided the number of *Wolbachia* genomes relative to the *Ae*. *albopictus* genomes. We measured the density of *Wolbachia* in individuals (N = 75) classified according to their IDT scores (0, 1, 2 or 3) defined as follows: the IDT score 0 for mosquitoes with no infectious DENV-1 particles either in the body, head or saliva; the IDT score 1 for samples with only infected bodies; the IDT score 2 for mosquitoes with infectious particles in the bodies and the heads; and the IDT score 3 for mosquitoes with infectious DENV-1 particles in the bodies, heads and saliva. dpe = days post-exposure.
(DOC)
