# Supplementary material for: Vector competence of Aedes albopictus field populations from Reunion Island exposed to local epidemic dengue viruses
Source: PLoS One. 2024 Sep 19;19(9):e0310635. doi: 10.1371/journal.pone.0310635 (PMC11412507; doi:10.1371/journal.pone.0310635)
Supplement: S1 Table — The IDT (Infection, Dissemination, Transmission) score (0, 1, 2 or 3) was defined as follows: the IDT score 0 for mosquitoes with no infectious DENV-1 particles either in the body, head or saliva; the IDT score 1 for samples with only infected bodies; the IDT score 2 for mosquitoes with infectious particles in the bodies and the heads; and the IDT score 3 for mosquitoes with infectious DENV-1 particles in the bodies, heads and saliva. These categories were defined using mosquitoes exposed to DENV-1 and collected 21 and 28 dpe. Mosquitoes were from four populations: F0_SM (Sainte-Marie), F0_SG (Saint-Gilles les Hauts), F0_SP (Saint-Philippe) and F0_SA (Saint-André). (DOC) [file pone.0310635.s001.doc]

**S1 Table.**

|  | **21 dpe** | | | | **28 dpe** | | | |  |
| --- | --- | --- | --- | --- | --- | --- | --- | --- | --- |
| **IDT score** | **F0_SM** | **F0_SG** | **F0_SPh** | **F0_SA** | **F0_SM** | **F0_SG** | **F0_SPh** | **F0_SA** | **Total** |
| **0** | 3 | 3 | 3 | 3 | 2 | 3 | 3 | 3 | **23** |
| **1** | 1 | 2 | 3 | 1 | 0 | 3 | 1 | 1 | **12** |
| **2** | 3 | 4 | 3 | 3 | 3 | 3 | 2 | 0 | **21** |
| **3** | 3 | 2 | 1 | 3 | 3 | 3 | 3 | 1 | **19** |
| **Total** | **10** | **11** | **10** | **10** | **8** | **12** | **9** | **5** | **75** |
