# Supplementary material for: Vector competence of Aedes albopictus field populations from Reunion Island exposed to local epidemic dengue viruses
Source: PLoS One. 2024 Sep 19;19(9):e0310635. doi: 10.1371/journal.pone.0310635 (PMC11412507; doi:10.1371/journal.pone.0310635)
Supplement: S2 Table — Infection rates (IR), dissemination efficiencies (DE), and transmission efficiencies (TE) were examined at 14 and 21 days post-exposure (dpe) to an infectious blood meal. IR = number of infected bodies among the mosquitoes tested (%); DE = number of infected heads among the mosquitoes tested (%); TE = number of infected saliva among the mosquitoes tested (%). The numbers in brackets correspond to the 95% confidence interval, and the numbers in parentheses represent the number of positive samples out of the total number of samples tested. ND = not done. F1_SPa, F1_SL and F1_LP correspond to Ae. albopictus populations and F31_Aeg is the Ae. aegypti population. (DOC) [file pone.0310635.s002.doc]

**S2 Table.**

| **Population** | **14 dpe** | | | **21 dpe** | | |
| --- | --- | --- | --- | --- | --- | --- |
|  | **IR** | **DE** | **TE** | **IR** | **DE** | **TE** |
| **F1_SPa** | 11.76%  [3.29 - 34.34%]  (2/17) | 0.00%  [0.00 - 18.43%]  (0/17) | 0.00%  [0.00 - 18.43%]  (0/17) | ND | ND | ND |
| **F1_SL** | 9.09%  [2.53 - 27.81%]  (2/22) | 0.00%  [0.00 - 14.87%]  (0/22) | 0.00%  [0.00 - 14.87%]  (0/22) | ND | ND | ND |
| **F1_LP** | 5.88%  [1.05 - 26.98%]  (1/17) | 0.00%  [0.00 - 18.43%]  (0/17) | 0.00%  [0.00 - 18.43%]  (0/17) | ND | ND | ND |
| **F31_Aeg** | 9.38%  [3.24 - 24.22%]  (3/32) | 0.00%  [0.00 - 10.72%]  (0/32) | 0.00%  [0.00 - 10.72%]  (0/32) | 8.82%  [3.05 - 22.96%]  (3/34) | 0.00%  [0.00 - 10.15%]  (0/34) | 0.00%  [0.00 - 10.15%]  (0/34) |
