# Supplementary material for: Vector competence of Aedes albopictus field populations from Reunion Island exposed to local epidemic dengue viruses
Source: PLoS One. 2024 Sep 19;19(9):e0310635. doi: 10.1371/journal.pone.0310635 (PMC11412507; doi:10.1371/journal.pone.0310635)
Supplement: S3 Table — Infection rates (IR), dissemination efficiencies (DE), and transmission efficiencies (TE) were examined at 14, 21, and 28 days post-exposure (dpe) to an infectious blood meal. IR = number of infected bodies among the mosquitoes tested (%); DE = number of infected heads among the mosquitoes tested (%); TE = number of infected saliva among the mosquitoes tested (%). The numbers in brackets correspond to the 95% confidence interval, and the numbers in parentheses represent the number of positive samples out of the total number of samples tested. ND = not done. F0_SM, F0_SA, F0_SG and F0_SPh correspond to Ae. albopictus populations and F31_Aeg is the Ae. aegypti population. (DOC) [file pone.0310635.s003.doc]

**S3 Table.**

| **Population** | **14 dpe** | | | **21 dpe** | | | **28 dpe** | | |
| --- | --- | --- | --- | --- | --- | --- | --- | --- | --- |
|  | **IR** | **DE** | **TE** | **IR** | **DE** | **TE** | **IR** | **DE** | **TE** |
| **F0_SM** | 2.08%  [0.37 - 10.90%]  (1/48) | 0.00 %  [0.00 - 7.41%] (0/48) | 0.00 %  [0.00 - 7.41%] (0/48) | 0.00 %  [0.00 - 7.41%] (0/48) | 0.00 %  [0.00 - 7.41%]  (0/48) | 0.00 %  [0.00 - 7.41%]  (0/48) | 0.00 %  [0.00 - 7.41%]  (0/48) | 0.00 %  [0.00 - 7.41%]  (0/48) | 0.00 %  [0.00 - 7.41%]  (0/48) |
| **F0_SA** | 2.08%  [0.37 - 10.90%] (1/48) | 0.00 %  [0.00 - 7.41%]  (0/48) | 0.00 %  [0.00 - 7.41%]  (0/48) | 0.00 %  [0.00 - 7.41%]  (0/48) | 0.00 %  [0.00 - 7.41%]  (0/48) | 0.00 %  [0.00 - 7.41%]  (0/48) | 0.00 %  [0.00 - 7.41%]  (0/48) | 0.00 %  [0.00 - 7.41%]  (0/48) | 0.00 %  [0.00 - 7.41%]  (0/48) |
| **F0_SG** | ND | ND | ND | ND | ND | ND | 0.00 %  [0.00 - 43.45%]  (0/5) | 0.00 %  [0.00 - 43.45%] (0/5) | 0.00 %  [0.00 - 43.45%]  (0/5) |
| **F0_SPh** | ND | ND | ND | ND | ND | ND | 25.00%  [10.18 - 49.50%] (4/16) | 0.00 %  [0.00 - 19.36%] (0/16) | 0.00 %  [0.00 - 19.36%]  (0/16) |
| **F31_Aeg** | 8.57%  [2.96 - 22.38%] (3/35) | 2.86%  [0.51 - 14.53%] (1/35) | 0.00%  [0.00 - 9.89%] (0/35) | ND | ND | ND | ND | ND | ND |
