# Supplementary material for: Vector competence of Aedes albopictus field populations from Reunion Island exposed to local epidemic dengue viruses
Source: PLoS One. 2024 Sep 19;19(9):e0310635. doi: 10.1371/journal.pone.0310635 (PMC11412507; doi:10.1371/journal.pone.0310635)
Supplement: S4 Table — All dpe were tested independently. The comparison of the vector competence parameters (IR, DE, or TE) were performed with pairwise proportion comparison tests for each parameter and each dpe independently. The numbers in brackets correspond to the 95% confidence interval, and the numbers in parentheses represent the number of positive samples out of the total number of samples tested. Only comparisons with a significant difference (P < 0.05) are presented in the table. (DOC) [file pone.0310635.s004.doc]

**S4 Table.**

| **Variable** | **Day post-exposure** | **Population 1** | | **Population 2** | | **p-value<0.05 pairwise proportion test** |
| --- | --- | --- | --- | --- | --- | --- |
|  |  | **Population code** | **Variable value** | **Population code** | **Variable value** |  |
| **IR** | **14 dpe** | **F2_BP** | 64.58%  [50.44 - 76.57%] (31/48) | **F0_SG** | 9.38% [3.24 - 24.22%] (3/32) | **6.55E-05** |
|  |  |  |  | **F0_SPh** | 15.63% [6.86 - 31.75%] (5/32) | **9.35E-04** |
|  |  | **F2_TB** | 54.17%  [40.29 - 67.42%] (26/48) | **F0_SG** | 9.38% [3.24 - 24.22%] (3/32) | **2.53E-03** |
|  |  |  |  | **F0_SPh** | 15.63% [6.86 - 31.75%] (5/32) | **2.58E-02** |
|  | **21 dpe** | **F2_BP** | 68.75%  [54.67 - 80.05%] (33/48) | **F0_SA** | 18.75% [10.19 - 31.94%] (9/48) | **4.67E-05** |
|  |  |  |  | **F0_SM** | 27.08% [16.57 - 41.00%] (13/48) | **2.18E-03** |
|  |  | **F2_SC** | 52.63%  [37.26 - 67.52%] (20/38) | **F0_SA** | 18.75% [10.19 - 31.94%] (9/48) | **4.48E-02** |
|  | **28 dpe** | **F2_BP** | 57.14%  [45.48- 68.06%] (40/70) | **F0_SA** | 9.38% [3.24 - 24.22%] (3/32) | **2.37E-04** |
|  |  |  |  | **F0_SM** | 20.83% [11.73- 34.26%] (10/48) | **2.86E-03** |
|  |  |  |  | **F0_SPh** | 14.58% [7.25 - 27.17%] (7/48) | **1.30E-04** |
|  |  | **F2_TB** | 68.42%  [57.30- 77.77%] (52/76) | **F0_SA** | 9.38% [3.24 - 24.22%] (3/32) | **1.03E-06** |
|  |  |  |  | **F0_SM** | 20.83% [11.73- 34.26%] (10/48) | **9.64E-06** |
|  |  |  |  | **F0_SPh** | 14.58% [7.25 - 27.17%] (7/48) | **2.24E-07** |
|  |  |  |  | **F0_SG** | 31.25% [19.95- 45.33%] (15/48) | **1.70E-03** |
| **DE** | **21 dpe** | **F2_BP** | 64.58%  [50.44 - 76.57%] (31/48) | **F0_SA** | 16.67% [8.70 - 29.58%] (8/48) | **1.01E-04** |
|  |  |  |  | **F0_SM** | 25.00% [14.92 - 38.78%] (12/48) | **4.63E-03** |
|  |  |  |  | **F0_SPh** | 12.50% [5.86 - 24.70%] (6/48) | **1.01E-05** |
|  |  |  |  | **F0_SG** | 12.50% [5.86 - 24.70%] (6/48) | **1.01E-05** |
|  |  | **F2_TB** | 45.83%  [32.58 - 59.71%] (22/48) | **F0_SPh** | 12.50% [5.86 - 24.70%] (6/48) | **1.59E-02** |
|  |  |  |  | **F0_SG** | 12.50% [5.86 - 24.70%] (6/48) | **1.59E-02** |
|  | **28 dpe** | **F2_BP** | 55.71%  [44.08- 66.75%] (39/70) | **F0_SA** | 6.25% [1.73 - 20.15%] (2/32) | **9.72E-05** |
|  |  |  |  | **F0_SM** | 20.83% [11.73- 34.26%] (10/48) | **5.02E-03** |
|  |  |  |  | **F0_SPh** | 12.50% [5.86 - 24.70%] (6/48) | **7.87E-05** |
|  |  |  |  | **F0_SG** | 12.50% [5.86 - 24.70%] (6/48) | **7.87E-05** |
|  |  | **F2_TB** | 67.11%  [55.94- 76.62%] (51/76) | **F0_SA** | 6.25% [1.73 - 20.15%] (2/32) | **3.91E-07** |
|  |  |  |  | **F0_SM** | 20.83% [11.73- 34.26%] (10/48) | **1.99E-05** |
|  |  |  |  | **F0_SPh** | 12.50% [5.86 - 24.70%] (6/48) | **1.28E-07** |
|  |  |  |  | **F0_SG** | 12.50% [5.86 - 24.70%] (6/48) | **1.28E-07** |
| **TE** | **21 dpe** | **F2_BP** | 35.42%  [23.43 - 49.56%] (17/48) | **F0_SPh** | 2.08% [0.37 - 10.90%] (1/48) | **1.84E-03** |
|  |  |  |  | **F0_SG** | 4.17% [1.15 - 13.98%] (2/48) | **7.04E-03** |
|  | **28 dpe** | **F2_BP** | 32.86%  [23.00- 44.50%] (23/70) | **F0_SA** | 3.13% [0.55 - 15.74%] (1/32) | **3.63E-02** |
|  |  |  |  | **F0_SG** | 6.25% [2.15- 16.84%] (3/48) | **2.06E-02** |
|  |  | **F2_TB** | 40.79%  [30.44- 52.02%] (31/76) | **F0_SA** | 3.13% [0.55 - 15.74%] (1/32) | **3.45E-03** |
|  |  |  |  | **F0_SM** | 12.50% [5.86 - 24.70%] (6/48) | **2.43E-02** |
|  |  |  |  | **F0_SPh** | 8.33% [3.29- 19.55%] (4/48) | **3.15E-03** |
|  |  |  |  | **F0_SG** | 6.25% [2.15- 16.84%] (3/48) | **9.79E-04** |
