# Supplementary material for: Vector competence of Aedes albopictus field populations from Reunion Island exposed to local epidemic dengue viruses
Source: PLoS One. 2024 Sep 19;19(9):e0310635. doi: 10.1371/journal.pone.0310635 (PMC11412507; doi:10.1371/journal.pone.0310635)
Supplement: S5 Table — Infection rates (IR), dissemination efficiencies (DE), and transmission efficiencies (TE) were examined at 14, 21, and 28 days post-exposure (dpe) to an infectious blood meal. IR = number of infected bodies among the mosquitoes tested (%); DE = number of infected heads among the mosquitoes tested (%); TE = number of infected saliva among the mosquitoes tested (%). The numbers in brackets correspond to the 95% confidence interval, and the numbers in parentheses represent the number of positive samples out of the total number of samples tested. ND = not done. F0_SM and F0_SM-bis correspond to Ae. albopictus populations of Sainte-Marie collected on the field in April and May 2021, respectively. (DOC) [file pone.0310635.s005.doc]

**S5 Table.**

| **Population** | **14 dpe** | | | **21 dpe** | | | **28 dpe** | | |
| --- | --- | --- | --- | --- | --- | --- | --- | --- | --- |
|  | **IR** | **DE** | **TE** | **IR** | **DE** | **TE** | **IR** | **DE** | **TE** |
| **F0_SM** | **28.13%**  **[15.56 - 45.37%]**  **(9/32)** | **3.13%**  **[0.55 - 15.74%]**  **(1/32)** | **3.13%**  **[0.55 - 15.74%]**  **(1/32)** | **27.08%**  **[16.57 - 41.00%]**  **(13/48)** | **25.00%**  **[14.92 - 38.78%]**  **(12/48)** | **14.58%**  **[7.25 - 27.17%]**  **(7/48)** | **20.83%**  **[11.73 - 34.26%]**  **(10/48)** | **20.83%**  **[11.73 - 34.26%]**  **(10/48)** | **12.50%**  **[5.86 - 24.70%]**  **(6/48)** |
| **F0_SM-bis** | **25.00%**  **[12.00 - 44.90%]**  **(6/24)** | 0.00%  [0.00 - 13.80%]  (0/24) | 0.00%  [0.00 - 13.80%]  (0/24) | **25.00%**  **[12.00 - 44.90%]**  **(6/24)** | **12.50%**  **[4.34 - 31.00%]**  **(3/24)** | **8.33%**  **[2.32 - 25.85%]**  **(2/24)** | **26.92%**  **[13.70 - 46.08%]**  **(7/26)** | **15.38%**  **[6.15 - 33.53%]**  **(4/26)** | **3.85%**  **[0.68 - 18.89%]**  **(1/26)** |
