# Supplementary material for: Vector competence of Aedes albopictus field populations from Reunion Island exposed to local epidemic dengue viruses
Source: PLoS One. 2024 Sep 19;19(9):e0310635. doi: 10.1371/journal.pone.0310635 (PMC11412507; doi:10.1371/journal.pone.0310635)
Supplement: S6 Table — The mosquitoes of F0 generation, belonging to the populations of Sainte-Marie (F0_SM), Saint-Gilles les Hauts (F0_SG), Saint-Philippe (F0_SPh) and Saint-André (F0_SA), were examined at 21 and 28 days after being exposed to infectious blood meals containing the DENV-1 strain. In this table N = number of mosquitoes tested; 95% CI, 95% confidence interval. wAlbTot = wAlbA + wAlbB. (DOC) [file pone.0310635.s006.doc]

**S6 Table.**

| **Population** | ***w*AlbA** | | ***w*AlbB** | | ***w*AlbTot** | |
| --- | --- | --- | --- | --- | --- | --- |
|  | **median** | **95% CI median** | **median** | **95% CI median** | **median** | **95% CI median** |
| **F0_SM** (N=18) | 4.75 | 2.20 - 11.22 | 1.95 | 1.40 - 2.30 | 6.60 | 4.20 - 12.94 |
| **F0_SG** (N=23) | 2.20 | 0.70 - 2.70 | 0.45 | 0.27 - 0.80 | 2.60 | 1.22 - 3.30 |
| **F0_SPh** (N=19) | 4.00 | 2.50 - 7.79 | 0.40 | 0.23 - 0.60 | 4.81 | 2.80 - 8.11 |
| **F0_SA** (N=15) | 9.00 | 3.90 - 24.80 | 1.80 | 1.00 - 4.70 | 12.80 | 6.91 - 26.90 |
