# Supplementary material for: Vector competence of Aedes albopictus field populations from Reunion Island exposed to local epidemic dengue viruses
Source: PLoS One. 2024 Sep 19;19(9):e0310635. doi: 10.1371/journal.pone.0310635 (PMC11412507; doi:10.1371/journal.pone.0310635)
Supplement: S7 Table — The mosquitoes of F0 generation, belonging to the populations of Sainte-Marie (F0_SM), Saint-Gilles les Hauts (F0_SG), Saint-Philippe (F0_SPh) and Saint-André (F0_SA) and examined at 21 and 28 days after being exposed to infectious blood meals containing the DENV-1 strain, were pooled according to their IDT scores (0, 1, 2 or 3). IDT scores are defined as follows: IDT score 0 for mosquitoes with no infectious DENV-1 particles either in the body, head or saliva; the IDT score 1 for samples with only infected bodies; the IDT score 2 for mosquitoes with infectious particles in the bodies and the heads; and the IDT score 3 for mosquitoes with infectious DENV-1 particles in the bodies, heads and saliva. In this table: N, number of mosquitoes tested; 95% CI, 95% confidence interval. (DOC) [file pone.0310635.s007.doc]

**S7 Table.**

| **Score IDT** | ***w*AlbA** | | ***w*AlbB** | | ***w*AlbTot** | |
| --- | --- | --- | --- | --- | --- | --- |
|  | **median** | **95% CI median** | **median** | **95% CI median** | **median** | **95% CI median** |
| **IDT 0**  (N=23) | 3.70 | 2.30 - 10.04 | 1.00 | 0.48 - 1.75 | 4.18 | 2.80 - 11.88 |
| **IDT 1**  (N=12) | 3.95 | 0.60 - 7.79 | 0.31 | 0.20 - 0.97 | 5.61 | 0.74 - 9.90 |
| **IDT 2**  (N=21) | 5.48 | 2.60 - 11.22 | 1.72 | 0.50 - 2.10 | 6.90 | 4.81 - 12.94 |
| **IDT 3**  (N=19) | 2.95 | 2.07 - 6.26 | 0.80 | 0.40 - 1.54 | 4.40 | 2.60 - 7.20 |
| **IDT 1+2+3**  (N=52) | 4.20 | 2.60 - 6.11 | 0.80 | 0.50 - 1.40 | 5.72 | 4.20 - 7.50 |
| **IDT 2+3**  (N=40) | 4.41 | 2.60 - 6.20 | 0.86 | 0.60 - 1.80 | 5.72 | 4.20 - 7.70 |
