# Supplementary material for: Vector competence of Aedes albopictus field populations from Reunion Island exposed to local epidemic dengue viruses
Source: PLoS One. 2024 Sep 19;19(9):e0310635. doi: 10.1371/journal.pone.0310635 (PMC11412507; doi:10.1371/journal.pone.0310635)
Supplement: S8 Table — The mosquitoes of F0 generation, belonging to the populations of Sainte-Marie (F0_SM), Saint-Gilles les Hauts (F0_SG), Saint-Philippe (F0_SPh), or Saint-André (F0_SA) and examined at 21 and 28 days post-exposure (dpe) to infectious blood meals containing the DENV-1 local strain. In this table: N, number of mosquitoes tested; sd, standard deviation; 95% CI, 95% confidence interval; med = median. (DOC) [file pone.0310635.s008.doc]

**S8 Table.**

| **Population** | **number of DENV-1 RNA copies** | | | | |
| --- | --- | --- | --- | --- | --- |
|  | **mean** | **sd** | **95% CI mean** | **med** | **95% CI med** |
| **F0_SM** (N=12) | 1.60x10^7^ | 1.09x10^7^ | 9.09x10^6^ - 2.30x10^7^ | 1.36x10^7^ | 6.78x10^6^ - 2.60x10^7^ |
| **F0_SG** (N=13) | 2.90x10^7^ | 3.92x10^7^ | 5.32x10^6^ - 5.28x10^7^ | 1.40x10^7^ | 3.41x10^1^ - 4.01x10^7^ |
| **F0_SPh** (N=9) | 3.95x10^5^ | 3.69x10^5^ | 1.11x10^5^ - 6.78x10^5^ | 2.72x10^5^ | 5.05x10^4^ - 7.71x10^5^ |
| **F0_SA** (N=9) | 9.58x10^6^ | 9.20x10^6^ | 2.51x10^6^ - 1.66x10^7^ | 4.89x10^6^ | 3.28x10^6^ - 1.46x10^7^ |
