# Supplementary figures and images for: Vector competence of Aedes albopictus field populations from Reunion Island exposed to local epidemic dengue viruses
Source: PLoS One. 2024 Sep 19;19(9):e0310635. doi: 10.1371/journal.pone.0310635 (PMC11412507; doi:10.1371/journal.pone.0310635)

**
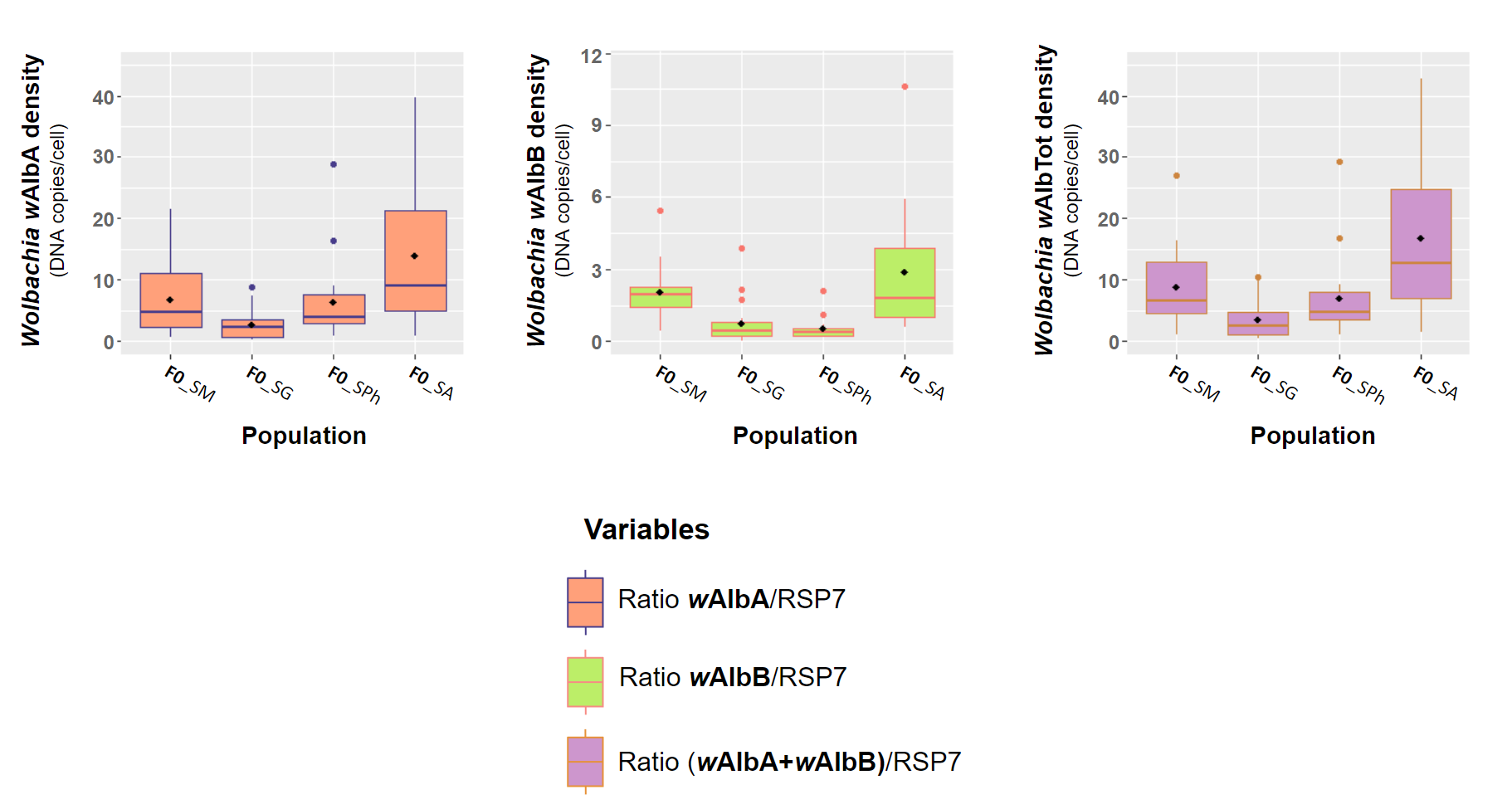
S2 Fig.**

Supplement: S2 Fig — The mosquitoes of F0 generation from four populations (N = 75) namely Sainte-Marie (F0_SM), Saint-Gilles les Hauts (F0_SG), Saint-Philippe (F0_SPh) and Saint-André (F0_SA) were tested for their Wolbachia densities after being exposed to infectious blood meals containing the DENV-1 strain. For each sample, the value provided corresponds to the mean of a triplicate measure. The densities of Wolbachia wAlbA (orange), or wAlbB (green), or wAlbTot (wAlbA + wAlbB) (violet), are given based on the ratio between the Wolbachia and RSP7 concentrations which provided the number of Wolbachia genomes relative to the Ae. albopictus genomes. dpe = days post-exposure. (DOC) [file pone.0310635.s010.doc]

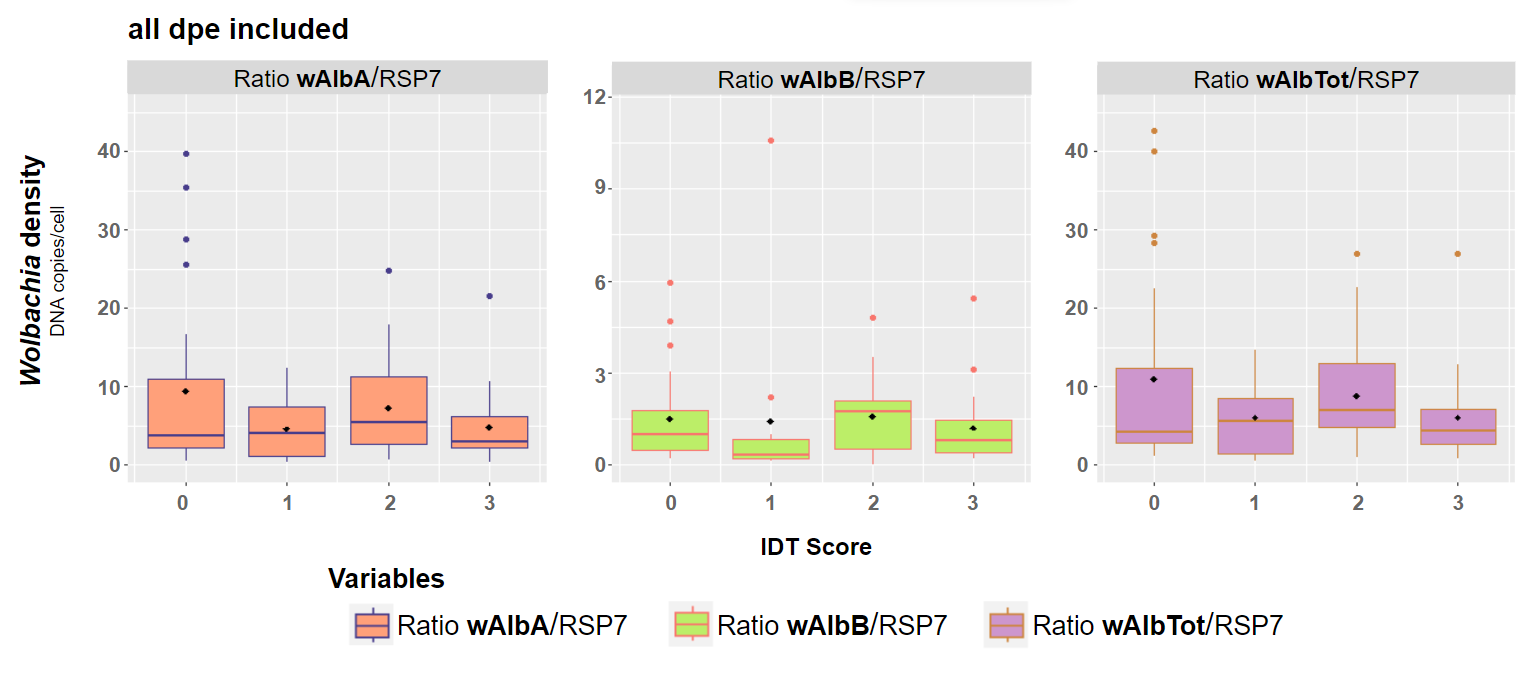


**S3 Fig.**

Supplement: S3 Fig — These analyses were performed using individual mosquito of F0 generation, belonging to the populations of Sainte-Marie (F0_SM), Saint-Gilles les Hauts (F0_SG), Saint-Philippe (F0_SPh), or Saint-André (F0_SA) and examined at 21 and 28 days after being exposed to infectious blood meals containing the DENV-1 strain. The densities of Wolbachia wAlbA (orange), or wAlbB (green), or wAlbTot (wAlbA + wAlbB) (violet) (violet) are given based on the ratio between the Wolbachia and RSP7 concentrations which provided the number of Wolbachia genomes relative to the Ae. albopictus genomes. We measured the density of Wolbachia in individuals (N = 75) classified according to their IDT scores (0, 1, 2 or 3) defined as follows: the IDT score 0 for mosquitoes with no infectious DENV-1 particles either in the body, head or saliva; the IDT score 1 for samples with only infected bodies; the IDT score 2 for mosquitoes with infectious particles in the bodies and the heads; and the IDT score 3 for mosquitoes with infectious DENV-1 particles in the bodies, heads and saliva. dpe = days post-exposure. (DOC) [file pone.0310635.s011.doc]
